# CEBP-β and PLK1 as Potential Mediators of the Breast Cancer/Obesity Crosstalk: In Vitro and In Silico Analyses

**DOI:** 10.3390/nu15132839

**Published:** 2023-06-22

**Authors:** Felice Maria Accattatis, Amanda Caruso, Alfonso Carleo, Piercarlo Del Console, Luca Gelsomino, Daniela Bonofiglio, Cinzia Giordano, Ines Barone, Sebastiano Andò, Laura Bianchi, Stefania Catalano

**Affiliations:** 1 Department of Pharmacy, Health and Nutritional Sciences, Via P. Bucci, University of Calabria, Arcavacata di Rende (CS), 87036 Cosenza, Italy; 2 Department of Pulmonology, Hannover Medical School, Carl-Neuberg-Straße, 30625 Hannover, Germany; 3 Centro Sanitario, Via P. Bucci, University of Calabria, Arcavacata di Rende (CS), 87036 Cosenza, Italy; 4 Section of Functional Proteomics, Department of Life Sciences, Via Aldo Moro, University of Siena, 53100 Siena, Italy

**Keywords:** breast cancer, obesity, transcription factors, transcriptomics, bioinformatic functional processing, CEBP-β, PLK1

## Abstract

Over the last two decades, obesity has reached pandemic proportions in several countries, and expanding evidence is showing its contribution to several types of malignancies, including breast cancer (BC). The conditioned medium (CM) from mature adipocytes contains a complex of secretes that may mimic the obesity condition in studies on BC cell lines conducted in vitro. Here, we report a transcriptomic analysis on MCF-7 BC cells exposed to adipocyte-derived CM and focus on the predictive functional relevance that CM-affected pathways/processes and related biomarkers (BMs) may have in BC response to obesity. CM was demonstrated to increase cell proliferation, motility and invasion as well as broadly alter the transcript profiles of MCF-7 cells by significantly modulating 364 genes. Bioinformatic functional analyses unraveled the presence of five highly relevant central hubs in the direct interaction networks (DIN), and Kaplan–Meier analysis sorted the CCAAT/enhancer binding protein beta (CEBP-β) and serine/threonine-protein kinase PLK1 (PLK1) as clinically significant biomarkers in BC. Indeed, CEBP-β and PLK1 negatively correlated with BC overall survival and were up-regulated by adipocyte-derived CM. In addition to their known involvement in cell proliferation and tumor progression, our work suggests them as a possible “deus ex machina” in BC response to fat tissue humoral products in obese women.

## 1. Introduction

Overweight and obesity, as diseases of excessive fat deposition, are preventable conditions resulting from a chronic energy imbalance with dietary intake consistently exceeding expenditure over some period of time. Fueled by economic/social/technological changes, urbanization and nutritional transition, many countries have witnessed the prevalence of obesity rising at an alarming rate in the last decades. The World Obesity Atlas 2022, published by the World Obesity Federation, predicts that one billion people globally, including one in five women and one in seven men, will be living with obesity by 2030. This scenario has drawn significant attention from researchers since obesity has been associated with concomitant or increased odds of important chronic diseases, such as type 2 diabetes, dyslipidemia, stroke, cardiovascular disease, disability, and poor mental health [1]. Several studies have also highlighted an intricate connection between obesity and multiple types of malignancies, especially pancreatic, esophageal, colon, prostate, and breast cancers [2]. In particular, growing evidence demonstrates that excessive adiposity strongly impacts the incidence, prognosis and progression of breast carcinomas, with relevant implications for the clinical management of patients [3,4,5]. The relationship between overweight/obesity, BC and overall risk seems to rely on menopausal status, with obesity increasing BC risk in postmenopausal women but, conversely, being protective in premenopausal women [6,7,8]. However, a high body mass index (BMI) has been associated with larger tumor size, lymph node involvement, metastatic spread, and poorer survival outcomes in both pre- and post-menopausal women [9,10,11,12,13]. Various meta-analyses have also indicated a 35–40% increased risk of recurrence or death in obese breast cancer (BC) women compared with normal-weight patients [14,15,16]. Furthermore, a reduced effectiveness of anti-tumor adjuvant therapies, including radiotherapy, chemotherapy, and/or endocrine therapy, has also been evidenced among patients with a high BMI [3,4]. From a molecular perspective, adipocytes are progressively taking center stage in explaining the obesity–cancer link. Indeed, important adipocyte-derived mediators, such as adipokines, insulin-like growth factors, inflammatory cytokines, and metabolites, which are all abnormally modified in women affected by obesity, may interact with the intrinsic molecular characteristics of BC cells. This may result in the activation of several signaling pathways (e.g., MAPK and PI3K/Akt) and transcriptional factors (e.g., NF-κB and HIF-1α), thereby boosting BC progression and therapy resistance [4]. However, despite major advances in understanding these molecular “hits”, the intricate panorama of networks and cascades tying up obesity and BC needs still to be fully elucidated.

Here, we took advantage of an ‘in vitro’ model mimicking the adipocyte–BC cell interaction. The model was obtained by culturing MCF-7 BC cells with mature adipocyte-derived conditioned medium (CM), with the aim of delineating the impact that adipocyte secreted factors may have on the expression profile of BC cells. Our differential transcriptomic analysis evidenced MCF-7 cells as widely responsive BC cells to adipocyte CM. In addition, the MetaCore functional processing of the obtained results highlighted a tight functional correlation existing among genes significantly affected by the treatment, recognizing CCAAT/enhancer binding protein beta (CEBP-β) and serine/threonine-protein kinase PLK1 (PLK1) as key players in mediating the interactions between adipocytes and mammary cancer cells. The predictive functional overview we propose offers new insights in BC epithelial/adipose cell cross-talk and suggests attractive biomarkers for future applications in the prevention and treatment of women affected by BC and obesity.

## 2. Materials and Methods

### 2.1. Reagents and Antibodies

The following reagents and antibodies were used: RED-OIL-O (04-220923, Bio-Optica, Milan, Italy), GAPDH (6C5, sc-47724, Santa Cruz Biotechnology, Dallas, TX, USA), PLK1 (4535s, Cell Signaling Technology, Danvers, MA, USA), pPLK1 (9062S, Cell Signaling Technology), CEBP-β (E2K1U, 43095s, Cell Signaling Technology), pCEBP-β (Thr235, 3084s, Cell Signaling Technology), IRDye 800CW Goat anti-Rabbit (#925-32211, LI-COR, Lincoln, NE, USA), and IRDye 680RD Goat anti-Mouse (#925-68070, LI-COR) secondary antibodies.

### 2.2. Cell Cultures

Human MCF-7 and MDA-MB-231 breast cancer cell lines, murine 3T3-L1 pre-adipocytes were purchased from the American Type Culture Collection (Manassas, VA, USA) and cultured following supplier instruction. To obtain mature adipocytes, 3T3-L1 cells were cultured in pre-adipocyte growth medium for 72 h and then in differentiation medium which contains 1 μg/mL bovine insulin (Sigma-Aldrich, St. Louis, MO, USA), 0.5 mM 3-Isobutyl-1-Methylxanthine (Sigma-Aldrich) and 1 μM Dexamethasone (Sigma-Aldrich) for 48 h. Subsequently, adipocytes were maintained in growth medium with 10% FBS and 1 μg/mL bovine insulin for 14 days. Cells were frozen following manufacturer’s recommendations, used and authenticated by short tandem repeat analysis (AmpFLSTR Profiler Plus PCR Amplification Kit, Applied Biosystems, Monza, Italy) at our Sequencing Core within 4 months after frozen aliquots recovery, and systematically tested for mycoplasma-negativity (MycoAlert, Lonza, Basilea, Switzerland).

### 2.3. Lipid Droplet Visualization

Lipid droplets in adipocytes were detected by using Oil Red O. Adipocytes were fixed with 2% (*v*/*v*) formaldehyde for 30 min at 37 °C, 5% CO_2_. Then, the formaldehyde was discarded, the cells were washed with PBS two times, and subsequently, the plates were air dried. The cells were incubated with Oil Red O solution (three parts 0.5% Oil-Red O (*w*/*v*) in 60% isopropyl alcohol and two parts water) for 30 min. Thereafter, Oil Red O staining solution was withdrawn, and the cells were rinsed with water and air-dried. To obtain the images of the stained lipid droplet, an Olympus microscope (Olympus, Tokyo, Japan) was employed. Aiming to measure the lipid content, the dye was extracted from the stained adipocytes by using 60% isopropanol and then quantified by measuring the absorbance at 405 nm. Lipid content was shown as a fold in comparison with the pre-adipocytes as 1-fold lipid formation.

### 2.4. Real-Time RT-PCR

Total RNA from cells was extracted by using the TRIzol reagent (Thermo Fisher Scientific, Waltham, MA, USA). A NanoDrop-1000 spectrophotometer was used to evaluate RNA quality, integrity, and concentration. Two micrograms of total RNA was reverse transcribed with the RETROscript Kit (Applied Biosystems, Monza, Italy), and gene expression was analyzed by real-time RT-PCR using a SYBR Green Universal PCR Master Mix (Bio-Rad, Hercules, CA, USA) in an iCycler iQ Detection System (Bio-Rad). The relative gene expression levels were assessed and calculated as previously described [17]. Each sample was normalized on 18S mRNA expression. Primers are listed in Supplementary Table S1.

### 2.5. Conditioned Medium Systems

To produce conditioned medium (CM) from mature adipocytes (3T3-L1 A), cells were incubated in a culture medium of DMEM containing 3% Charcoal-Stripped Fetal Bovine Serum (CS FBS, Gibco), which is frequently used in studies on hormone-responsive cancers to provide hormone-free cell culture conditions, 100 mg/mL of L-glutamate (Sigma Aldrich), and 1% pen/strep (Sigma Aldrich) for 36–48 h. Then, CM was collected, centrifuged to remove cellular debris, and used in respective experiments.

### 2.6. RNA Library Preparation and Sequencing

Total RNA was extracted from cells as described [18]. RNA concentration was determined with a NanoDropOne spectrophotometer and quality assessed with an Agilent TapeStation4200 (Agilent Technologies, Santa Clara, CA, USA). High-quality RNA from three independent purifications for each experimental point was used for library preparation. Indexed libraries were prepared from 1 μg/ea. of purified RNA with TruSeq Stranded mRNA Library Prep (Illumina, San Diego, CA, USA) following suppliers. Libraries were quantified using the TapeStation 4200 (Agilent Technologies) and Qubit fluorometer (Invitrogen Co., Waltham, MA, USA); then, they were pooled such that each index-tagged sample was present in equimolar amounts. The pooled samples were subjected to cluster generation and sequencing using an Illumina NovaSeq6000 (Illumina) in a 2 × 75 paired-end format. RNA sequencing data have been deposited in the EBI ArrayExpress database (http://www.ebi.ac.uk/arrayexpress, accessed on February 2023) with Accession Number GSE223853.

### 2.7. MetaCore Functional Analysis

The differentially expressed transcriptome dataset (*n* = 364 genes) was functionally processed by applying the MetaCore™ integrated software suite for functional analysis of experimental data (Clarivate Analytics, London, UK). The gene name list was imported into MetaCore and processed for functional enrichment by “Drug and Xenobiotic Metabolism Enzymes” and “Pathway Map” ontologies using the Functional Ontology Enrichment tool. Differentially expressed genes were also investigated via the MetaCore Network Building tool and by applying the direct interaction algorithm. This functionally crosslinks gene transcripts under processing by building networks of corresponding proteins (and non-coding RNA) that directly interact with each other, according to the manually curated MetaCore in-house database of physiological and pathological protein–protein and protein–nucleic acid interactions, signaling and metabolic pathways from the scientific literature. The resulting direct interaction networks (DIN) represent the interatomic core of the investigated process and are prioritized according to their statistical significance (*p* ≤ 0.001). Nets are then graphically visualized as nodes (proteins) and edges (interconnections among proteins) and named in relation to their five most significant factors, i.e., central hubs. Since they establish the highest number of interactions with the other components of the net, central hubs acquire crucial roles in defining the biochemical and molecular properties of the investigated systems. In fact, they are usually recognized as highly significant biomarkers for understanding the physiological and pathological studied states as well as for drug design and pharmacological attempts, as we previously widely proved [18,19,20,21].

### 2.8. Cell Growth Assays

#### 2.8.1. Trypan-Blue Cell Count Assay

MCF-7 cells were seeded at a confluence of 50.000 cells/well in 24-well plates and treated for 48 h with CM. Cells were harvested by trypsinization and incubated in a 0.5% trypan blue solution for 10 min at room temperature. Trypan blue negative cells were counted through a Countess^®^ II Automated Cell Counter at day 0 and after 48 h of treatment.

#### 2.8.2. MTT Assay

Cell viability was determined utilizing the 3-(4,5-dimethylthiazol-2-yl)-2,5-diphenyltetrazolium (MTT) assay. Cells (5000 cells/well) were grown in 96-well plates and exposed to treatments as indicated. MTT (2 mg/mL, Sigma Aldrich) was added to each well, and the plates were incubated for 2 h at 37 °C followed by medium removal and solubilization in 100 μL DMSO (Sigma Aldrich). The absorbance was measured at 570 nm.

#### 2.8.3. Soft Agar Growth Assay

Anchorage-independent growth assays were conducted as previously described [20]. Data represent 3 independent experiments carried out in triplicate.

### 2.9. Transmigration Assay

MCF-7 cells were seeded at a confluence of 20,000 cells/well in the upper compartments of Boyden Chamber (8 μm membranes, Corning Costar, Corning, NY, USA), and transmigration assay was performed as described [22].

### 2.10. Invasion Assay

The Matrigel-based invasion assay was performed in chambers (8 μm membranes, Corning Costar) coated with Matrigel (2 mg/mL, BD Biosciences, Franklin Lakes, NJ, USA) in 24-well plates. Cells were seeded (20,000 cells/well) into top transwell chambers, and regular growth medium was used in the lower chambers. After 8–12 h, invaded cells were quantified as reported for transmigration assays.

### 2.11. Survival Kaplan–Meier Analysis

Kaplan–Meier plots were generated using the Kaplan–Meier plotter online tool (https://kmplot.com/analysis/, accessed on 10 March 2023) with autoselected best cutoff chosen and no restriction analysis to subtypes or cohorts applied. The Kaplan–Meier survival graph, hazard ratio (HR) with 95% confidence intervals (CI) and log-rank *p*-value were calculated and plotted in R using Bioconductor packages as indicated [23].

### 2.12. Immunoblot Analysis

Cells were lysed in RIPA Buffer supplemented with protease inhibitors as previously described [24]. Equal quantities of protein loads were resolved by SDS-PAGE and transferred to nitrocellulose membranes by using a trans-blot turbo transfer system (Bio-Rad, Hercules, CA, USA). Nitrocellulose membranes were blocked with 5% milk in TBST, which was followed by incubation with primary and fluorescence-labeled secondary antibodies. Specifically, an anti-full length (LAP*)/LAP primary antibody (Ab) (1:1000 dilution) (#43095) and an anti-LAP*/LAP/LIP-pThr235 primary Ab (1:500 dilution) (#3084) were, respectively, used to detect total C/EBP-β and pC/EBP-β (Thr235). PLK1 (#4535s), and pPLK1 (#9062S) were used at 1:250 dilution. An anti-rabbit IgG secondary Ab was used at 1:10,000 dilution. GAPDH immunodetection was applied to ensure an equal loading of samples in each lane. A mouse monoclonal anti-GAPDH (sc-47724), 1:1000 diluted, was used as the primary Ab and an anti-mouse IgG, 1:10,000 diluted, was used as the secondary Ab. Odyssey FC (Licor, Lincoln, NE, USA) and Image Studio Lite software were employed to, respectively, acquire and quantify the band of interest.

### 2.13. Statistical Analysis

Data were analyzed for statistical significance using a two-tailed Student’s *t* test performed by the GraphPad-Prism7 software program (GraphPad Inc., San Diego, CA, USA). *p* values less than 0.05 were considered significant. All data are shown as mean ± standard deviations (SD). For RNA sequencing bioinformatics analysis, Fastq underwent Quality Control using the FastQC tool (http://www.bioinformatics.babraham.ac.uk/projects/fastqc/, accessed on 15 February 2022). The tool cutadapt (version 2.5) was used to remove the adapter sequence and the very short reads (reads length < 20). The mapping of paired-end reads was performed using STAR (version 2.7.5c) with the standard parameters for paired reads on reference genome assembly hg38 obtained from GenCode (Release 37 (GRCh38.p13). The quantification of transcripts expressed for each sample was performed using the FeatureCount (version 2.0) [25] algorithm. DESeq2 [26] was used to perform the normalization matrix, and differentially expressed gene of all samples were considered. The overall comparative trend between the two investigated conditions was represented by a volcano plot. The transcriptomic values of genes, defined as differentially expressed genes (DEGs), presenting a BH-FDR ≤ 0.01 and an absolute log2FC higher than 2 were selected for further functional analyses and heatmap images. In particular, DEGs were clustered according to Ward’s method of the scaled Euclidean distances in the heatmap.

## 3. Results

### 3.1. Transcriptomic Profile Deregulation in MCF-7 BC Cells Exposed to Adipocyte CM and Predictive Functional Analysis of the Differentially Expressed Genes

**Figure 1:**
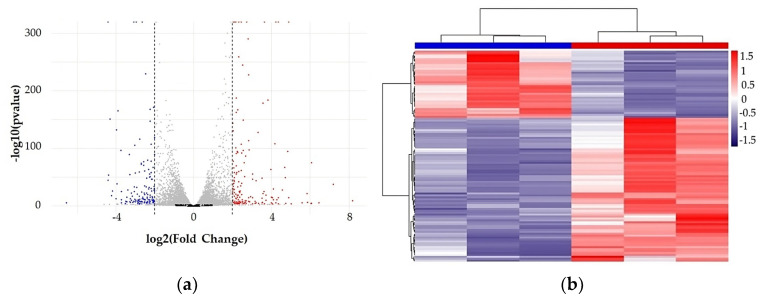
(**a**) Volcano plot representing all the detected mRNA (18.617 transcripts). The selected significant variables (364) are localized in the two lateral areas of the plot: on the right, genes up-regulated (red dots) in CM-treated MCF-7 and, on the left, genes down-regulated (blue dots) in CM-treated MCF-7 cells. Controls were treated with DMEM 3% CS-FBS (as basal conditions). Gray dots correspond to filtered out transcripts, which are not significantly affected, according to the selected parameters, by the treatment. FDR values have been converted to −log10. (**b**) Scaled heatmap showing the expression profiles of the 364 significant differences detected between CM untreated (horizontal-dendrogram blue bar) and treated (horizontal-dendrogram red bar) MCF-7 cells.

The aim of the present work was to investigate the effects of the adipocyte-derived conditioned medium on tumor characteristics of MCF-7 BC cells. To achieve this goal, MCF-7 cells were treated for 48 h with conditioned medium (CM) derived from differentiated adipocytes 3T3-L1-A (Supplementary Figure S1), which is a widely used ‘in vitro’ model of white adipocytes. These adipocytes actually display several features of different adipocyte lineages, such as gene expression profiles and basal bioenergetics [27]. The transcript abundance differential analysis, performed between CM untreated (control) and CM-treated MCF-7 BC cells, showed a consistent transcriptional modulation induced by CM treatment. The stringent cutoffs we applied for statistics (FDR ≥ 0.01) and fold change (−2 ≥ log2FC ≥ 2) relevance allowed us to recognize 364 highly significant genes modulated by CM treatment (Supplementary Table S2). Of these, 191 were up-regulated and 173 were down-regulated in CM-treated MCF-7 cells, as shown in Figure 1.

**Figure 2:**
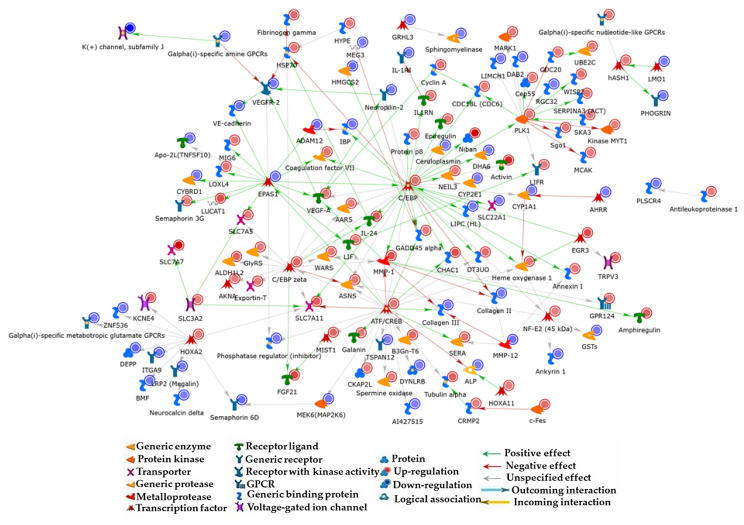
Direct interaction network built by processing DEGs detected between CM-treated MCF-7 and control untreated cells. Experimental DIN factors marked by a blue spot are down-regulated, whilst those marked by a red one are up-regulated. The intensity of the spot color is directly proportional to the deregulation level. Functions of different network items are identified by different symbols. Edge color and arrowheads indicate the type and the direction of protein interconnection. The green arrow indicates a positive effect, the red one indicates a negative effect, and the gray arrow indicates an unspecified interaction.

In order to predict biomarkers and/or related pathways for defining biochemical and molecular effects of adipose tissue on BC, we functionally processed the 364 significantly deregulated genes (DEGs) by applying the MetaCore network building tool and selecting the direct interaction algorithm. The resulting direct interaction network (DIN) included only experimental factors annotated as directly interacting in the MetaCore in-house database (Figure 2).

Thirty percent of DEGs, i.e., 111 experimental factors, codes for proteins that entered into the main DIN. Since transcriptomic profiles do not perfectly match with corresponding proteomic ones, this consistent inclusion of coded proteins, whose presence was predicted on the basis of the mRNA detection, into the same network strengthens the relevance of the obtained results and emphasizes the defined pathways as the interatomic core of CM effects on BC cells. CCAAT/enhancer binding protein beta (CEBP-β; group of C/EBP in the DIN), cyclic AMP-dependent transcription factor ATF-3 (ATF3; group of ATF/CREB in the DIN), DNA damage-inducible transcript 3 protein (DDIT3; CEBP zeta in the DIN), endothelial PAS domain-containing protein 1 (EPAS1), and serine/threonine-protein kinase polo-like kinase 1 (PLK1) were the main hubs with individual relevance corresponding to the order in which they are listed. With the sole exception of EPAS1, they are induced by the treatment in our transcriptomic analysis (Table 1).

CEBP-β is the fulcrum of network interactions, as it directly and indirectly correlates, through several experimental factors, to all the other four central hubs (Supplementary Figure S2). ATF3 seems also to play a relevant role in central hub interconnections by directly linking to CEBP-β, CEBP-zeta, and EPAS1. Moreover, the tight functional cross-talk existing among the five central hubs is further stressed by other CM-affected transcripts coding for proteins that act as molecular bridges for central hubs and on which up to three of the central hubs converge. Among these, we report: (i) CEBP-β, CEBP-zeta, and EPAS1 converging on vascular endothelial growth factor A (VEGF-A); (ii) CEBP-β and CEBP-zeta along with ATF3 on interstitial collagenase (MMP1), asparagine synthetase (glutamine-hydrolyzing) (ASNS), and cystine/glutamate transporter (SLC7A11); (iii) CEBP-β and ATF3 on DDIT3 upstream open reading frame protein (DT3UO), glutathione-specific gamma-glutamylcyclotransferase 1 (CHAC1), growth arrest and DNA damage-inducible protein GADD45 alpha (GADD45 alpha), and heme oxygenase 1 (HO-1); (iv) EPAS1 and ATF3 on leukemia inhibitory factor (LIF) and on protein phosphatase 1 regulatory subunit 3C (from the DIN group “phosphatase regulator (inhibitor)”; PTG); and (v) CEBP-β and CEBP-zeta on cytoplasmic alanine-tRNA ligase (AARS), interleukin (IL) 24, and endoplasmic reticulum chaperone BiP (from the DIN group HSP70; GRP78) (Supplementary Figure S2). Among these nodes, MMP-1, VEGFA, and CHAC1, which were up-regulated by the CM treatment, were negatively correlated with BC overall survival (OS), according to the Kaplan–Meier analysis (Supplementary Figure S3). While PLK1 is a protein kinase, CEBP-β, CEBP-zeta, EPAS1, and ATF/CREB are transcription factors (TF) whose CM-induced deregulation apparently impacts on the broad gene expression alterations indicated by our transcriptomics data. Since TF activity is often impaired in cancer and as several TF are considered suitable pharmacological targets [28], the identified DIN central hubs absolutely deserve attention in BC onset and progression in obese subjects. In addition, almost all the above-reported experimental factors acting as molecular bridges among the five central hubs are involved in critical processes for cancer development, including BC development [29,30,31,32]. Therefore, reciprocal regulation of the central hubs and their concerted action on the expression/function of the DIN nodes in which they converge (Supplementary Figure S2) could promote BC aggressiveness by cooperating within the obesity microenvironment.

### 3.2. Adipocyte-CM Affects MCF-7 BC Cell Growth, Motility and Invasion

**Figure 3:**
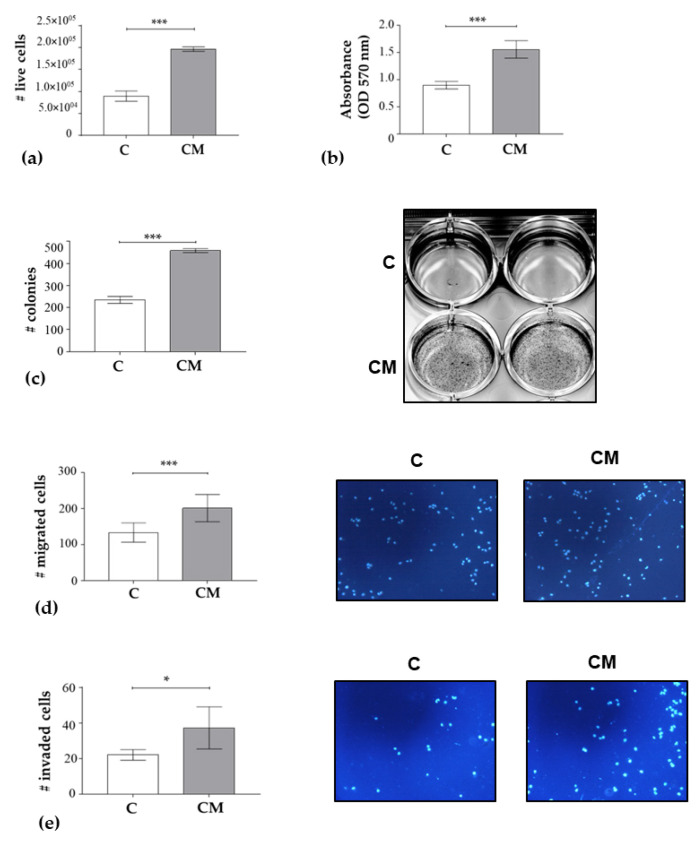
Impact of 3T3-L1 adipocyte-conditioned medium (CM) on MCF-7 cell phenotype. (**a**) Number of live cells in MCF-7 cells treated for 48 h with CM. Controls (C) were treated with DMEM 3% CS-FBS (basal conditions). Trypan blue negative cells were counted through a Countess^®^ II Automated Cell Counter at day 0 and after 48 h of treatment. (**b**) MTT assay performed 48 h after CM treatment. (**c**) Soft agar growth assays in MCF-7 cells treated or not with CM. After 14 days of growth, colonies ≥ 50 μm were counted. *Right panel*, representative pictures. (**d**) Transmigration and (**e**) invasion assays in MCF-7 cells treated as indicated. *Right panel*, representative pictures. * *p* < 0.05; *** *p* < 0.001.

As above mentioned, the central hubs of the DIN are involved in the control of the cell cycle. Since cancer cell uncontrolled proliferation is often associated with overacting cell cycle proteins and BC cellular aggressiveness is increased in obese patients, we evaluated whether CM may enhance MCF-7 cell growth in cell count, MTT, and anchorage-independent soft-agar growth assays (Figure 3a–c). We found that adipocyte-derived CM significantly increased the cell proliferation and colony numbers of MCF-7 cells. We also assessed if the adipocyte-CM treatment may affect MCF-7 cell motility and showed a significantly increased movement in transmigration assays (Figure 3d) as well as an enhanced capacity to invade an artificial membrane in invasion assays (Figure 3e).

Collectively, our data clearly indicated an enhanced cell proliferation, migration, and invasion due to CM treatment in MCF-7 BC cells.

### 3.3. Increased CEBP-β, and PLK1 Levels Correlate with a Shorter Overall Survival in BC Patients

**Figure 4:**
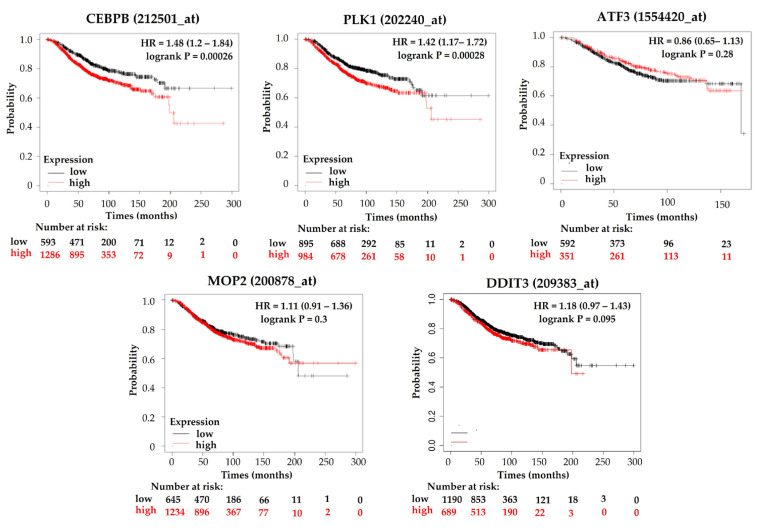
Kaplan–Meier survival analysis correlating the levels of CEBP-β, PLK1, ATF3, MOP2 (EPAS1) or CEPB-zeta (DDIT3) and overall survival (OS) in BC patients. Kaplan–Meier survival graph and hazard ratio with 95% confidence intervals and log-rank *p* value are shown.

In order to evaluate the potential clinical relevance of the DIN central hubs, retrospective analyses of the correlation among CEBP-β, PLK1, ATF3, EPAS1, and CEPB-zeta (DDIT3) levels and overall survival (OS) in patients with BC were conducted (Figure 4). Kaplan–Meier survival curves indicated that increased expression levels of CEBP-β and PLK1 are associated with a statistically significant shorter OS when compared with those tumors having low expression of these genes (CEBP-β: HR = 1.48, *p* = 0.00026; PLK1: HR = 1.42, *p* = 0.00028). In contrast, no significant difference was observed in the OS of BC patients in relation to ATF3, EPAS1 or CEBP-zeta expression levels. Thus, CEBP-β and PLK1 were selected for further analysis.

### 3.4. Effects of 3T3-L1 Adipocyte-CM on CEBP-β in BC Cells

**Figure 5:**
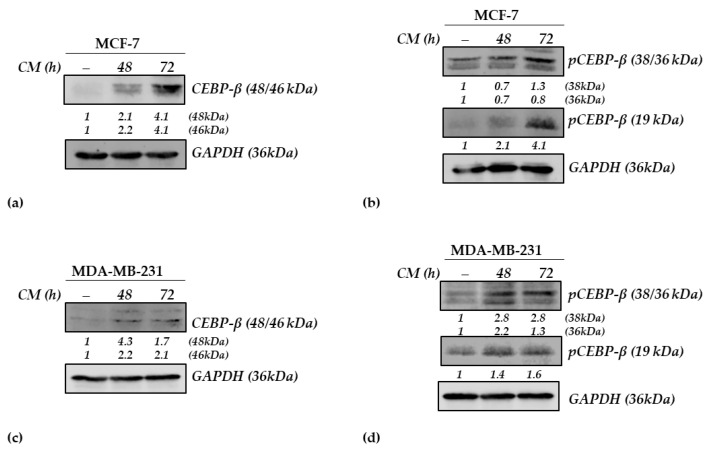
Effects of 3T3-L1 adipocyte-derived conditioned medium (CM) on CEBP-β phosphorylation and expression in BC cells. Immunoblotting showing (**a**) total protein CEBP-β and (**b**) phosphorylated (p) CEBP-β levels in MCF-7 BC cells after 48 and 72 h exposure to CM. Controls (−) were treated with DMEM 3% CS-FBS (basal conditions). Immunoblotting showing (**c**) total protein CEBP-β and (**d**) phosphorylated (p) CEBP-β levels in MDA-MB-231 BC cells treated as indicated. GAPDH was used as a control for equal loading and transfer. Italicized numbers below the blots represent the mean of the band optical density expressed as fold over CM-untreated cells.

CEBP-β belongs to a leucine-zipper TF family [33], and it plays several roles in different physiological and pathological processes, such as energy metabolism, cell proliferation, and cell differentiation [34]. Three different CEBP-β isoforms exist: p38, p33, and p20, which are also known as LAP*, LAP, and LIP, respectively [32,35]. In cancer, CEBP-β is mainly activated by phosphorylation, which leads to its translocation to the nucleus [36]. Based on this evidence and given the gene expression profile identified in our transcriptomic study, we evaluated the effects of CM on CEBP-β at the protein level (Figure 5).

Immunoblotting analysis demonstrated that CEBP-β LAP* and LAP isoform expression increased after 48 and 72 h of CM treatment (~2 and ~4 fold change, respectively). No signal was detected for LIP by using the Ab not specific for the proteoform of LIP. In addition, phosphorylation levels of the CEBP-β (pCEBP-β) LAP* isoform located at 38 kDa increased after 72 h of treatment, while the LAP isoform at 36 kDa remained stable. Interestingly, the pCEBP-β LIP isoform, at 19 kDa, rapidly increases after 48 and mostly after 72 h of CM treatment, reaching a 4.1 fold signal increase if compared to control.

We also performed immunoblotting analysis in a MDA-MB-231 triple-negative BC cell line treated for 48 and 72 h with adipocyte CM. As shown in Figure 5, CM treatment leads to an increase in CEBP-β expression at both total and phosphorylated levels. After analyzing the results we obtained in both ERα-positive and -negative cells, we can speculate that the induction of CEBP-β may be considered relevant in the biomolecular events that increase the aggressiveness of different kinds of BC cells exposed to adipose tissue products.

**Figure 6:**
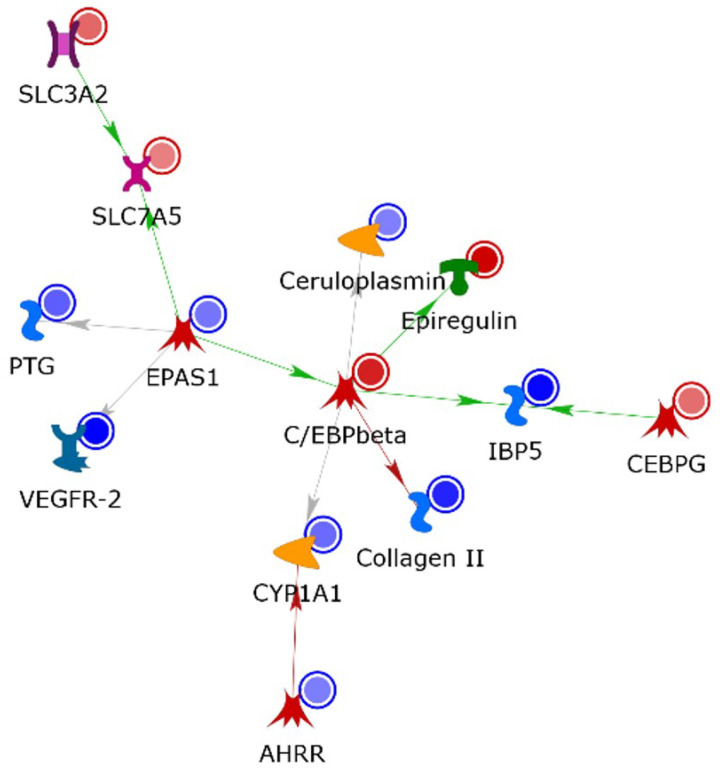
Direct interaction network built by processing the twenty-eight CM-DEGs annotated by the significantly enriched (FDR 1.07 × 10^−12^) term “AhR mediated regulation(_heart)“ in the MetaCore “Drug and Xenobiotic Metabolism Enzymes” ontology. Edge color and arrowheads indicate the type and the direction of protein interconnection. The green arrow indicates a positive effect, the red one indicates a negative effect, and the gray arrow indicates an unspecified interaction.

Interestingly, the MetaCore enrichment analysis in the “Drug and Xenobiotic Metabolism Enzymes” ontology highlighted a consistent significant enrichment (FDR 1.07 × 10^−12^) of genes involved in “AhR mediated regulation(_heart)”. Overall, 28 of the 364 deregulated genes we detected (Supplementary Table S3) were actually involved in this process, and 46% of them were directly linked (Figure 6). The deregulation of the AhR pathway was further stressed as occurring in adipocyte-CM exposed MCF-7 cells by the “AhR mediated regulation(_heart)” SPN, where all the twenty-eight enriched genes were included by adding single not-experimental factors (Supplementary Figure S4). As for the principal DIN built by processing the 364 DEGs, the AhR-mediated regulation nets, DIN and SPN, are centered on CEBP-β. Since both CEBP-β and AhR are shown to drive tumor progression [37,38,39,40], these findings provide important additional pathways that can be affected by adipocyte-derived CM.

### 3.5. Effects of 3T3-L1 Adipocyte-CM on PLK1 in BC Cells

**Figure 7:**
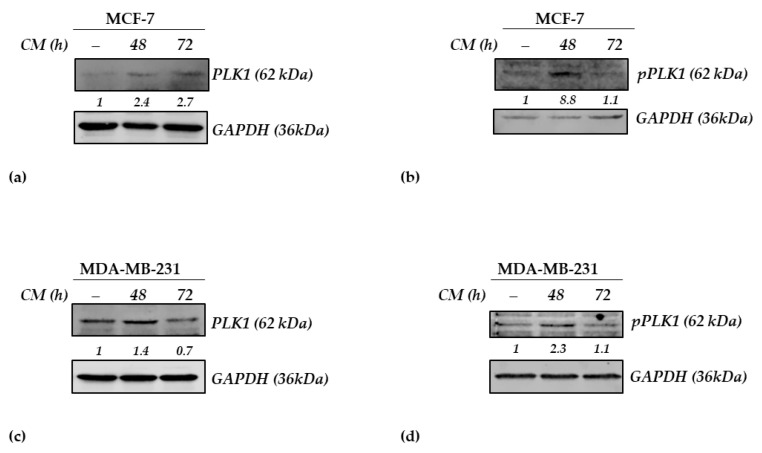
Effects of 3T3-L1 adipocyte-derived conditioned medium (CM) on PLK1 expression and phosphorylation in BC cells. Immunoblotting showing total protein PLK1 (**a**) and phosphorylated (p) PLK1 (**b**) levels in MCF-7 BC cells after 48 and 72 h exposure to conditioned medium. Immunoblotting showing total protein PLK1 (**c**) and phosphorylated (p) PLK1 (**d**) levels in MDA-MB-231 BC cells treated as indicated. GAPDH was used as a control for equal loading and transfer. Italicized numbers below the blot represent the mean of the band optical density expressed as fold over CM-untreated BC cells.

In vitro and in vivo studies proved that the serine/threonine protein kinase PLK1, whose phosphorylation is directly related to the kinase activity [41] is significantly correlated with metastasis, drug resistance, p53 mutation and stemness in BC cells [30,42,43,44,45,46]. To confirm RNA-sequencing results, we evaluated the effects of CM on the expression and phosphorylation levels of PLK1 in immunoblotting analysis. We found that exposure to adipocyte-derived CM leads to an increased abundance of the total and phosphorylated isoforms of PLK1 in MCF-7 cells, especially after 48 h of treatments (Figure 7a,b). Similar results were obtained in the triple-negative MDA-MB-231 BC cell line (Figure 7c,d).

**Figure 8:**
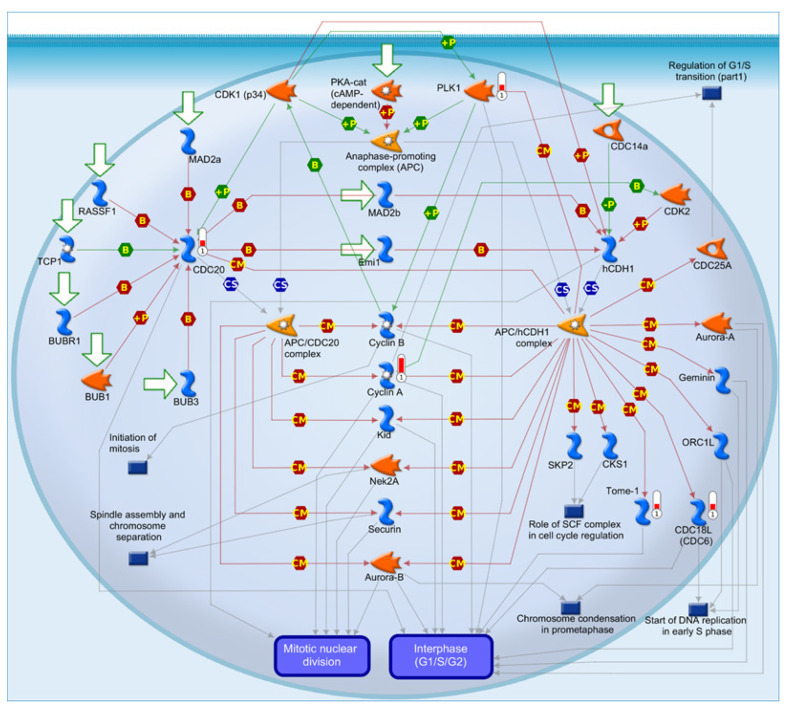
The MetaCore APC (anaphase-promoting complex) cell cycle regulation pathway. The top-scored regulated pathway may be supposed to be activated by treatment with CM in MCF-7 cells. Columns near experimental factors, i.e., PLK1, CDC18L (CDC6), Tome-1, and CDC20, show corresponding gene transcript up-regulation, which is proportional to the red mark height. Edge color and arrowheads indicate the type and the direction of protein interconnection. The green arrow indicates a positive effect, the red one indicates a negative effect, and the gray arrow indicates an unspecified interaction.

Moreover, the bioinformatic processing of transcriptomic results showed that CM-treated MCF-7 cells exhibited a significant enrichment (FDR: 0.02) of four factors annotated with the term “Cell cycle_role of APC in cell cycle regulation” from the MetaCore Pathway Map Ontology. Figure 8 shows how PLK1 is involved in this pathway, which is tightly related to anaphase-promoting complex (APC), which is a complex associated with tumorigenesis and progression in BC [47].

## 4. Discussion

Obesity represents a complex disorder which is primarily due to an excess of body fat. In addition to being an aesthetic problem, obesity participates in the risk, progression and prognosis of various diseases, including BC. Particularly, the correlation between excessive adiposity and cancer incidence, together with poor clinical outcome, has been already extensively described and discussed (reviewed in [4]). Here, we report a transcriptomic analysis on MCF-7 BC cells exposed to adipocyte-derived CM and focus on the predictive functional relevance of CM-affected pathways/processes.

### 4.1. Adipocyte-Derived CM Underscores a Specific Network of Protein Interconnection

The gene expression profile indicated the regulation of several transcriptional programs induced by CM treatment. In particular, the direct interaction network built by processing DEGs detected between CM-treated MCF-7 and -untreated cells evidenced the following five main hubs: CEBP-β, ATF3, DDIT3, CEBP zeta, EPAS1, and PLK1, with CEBP-β acting as the fulcrum of network interactions. Among regulatory factors, TFs from the CAAT enhancer family, as CEBP-β, play pivotal roles in adipocyte differentiation [34] and cancer progression [32]. In particular, CEBP-β is considered to be a potential candidate for therapeutic intervention in epithelial cancers because of its role in the control of cell proliferation, differentiation, and malignant transformation [32]. CEBP-β is a key regulator of mammary gland development and has been shown to control and influence proliferation and differentiation through different mechanisms in normal healthy tissue [39]. Interestingly, it has been demonstrated that this TF is able to transform normal mammary epithelial cells and induces epithelial to mesenchymal transition in cell culture, since CEBP-β overexpressing MCF10A cells acquired malignant phenotypic tracts, higher migration capacity, and an invasive phenotype [38]. Recently, it emerged as a regulator of the malignant BC behavior via Thrombospondin-2 (THBS2), whose expression is higher in invasive BC [40]. On the other hand, allelic variants in CEBP-β have been reported to influence abdominal obesity and related metabolic abnormalities in White Northern European families [48]. In addition, the transcription factor C/EBP-homologous protein (CHOP) (encoded by *DDIT3*), mainly induced by endoplasmic reticulum stress [49], is able to regulate numerous genes implicated in inflammation, differentiation, autophagy, and apoptosis [50,51,52]. Studies indicate that physiological conditions, including nutrient deprivation, DNA damage, and hypoxia, can induce CHOP expression [53], and the modulation of CHOP pathways may have a potential in treating cancers [54,55,56]. DDIT3 has been recently described as an essential mediator of death [57], as well as of tumoral growth suppression [58], and its deregulation promotes letrozole resistance [59] in BC cells. Interestingly, the DDIT3-rs697221 variant has been significantly associated with the risk of developing lung cancer [60], but BC is still not associated with this polymorphism.

ATF3 transcription factor is an adaptive-response gene involved in cellular processes to adapt extra and/or intracellular changes (i.e., inflammation, and metabolism) and oncogenesis by transducing signals from several receptors in order to either activate or repress gene expression [61,62,63,64]. Some studies suggest that ATF3 may be induced by several drugs, such as chemotherapeutic agents [65], including cisplatin [66], doxorubicin [67], and paclitaxel [68]. ATF3 expression is also stimulated by transforming growth factor-beta 1 (TGF-β1), and their interplay composes a positive feedback loop for TGF-β1 activation in BC cells [69]. Furthermore, the expression of ATF3 could mediate BC progression by the activation of pro-tumoral genes, such as cyclin A1, and the metastasis gene Runx2 [69]. ATF3 is also known as an activator of the tumor invasive potential by up-regulating the expression of the matrix metalloproteinase (MMP) family members (i.e., MMP-1, MMP2, MMP-9 and MMP-13) [70], whose mRNA and protein abundance are correlated with BC invasion and metastasis [71]. In line with these results, our analyses indicated MMP1 up-regulation in MCF-7 BC cells exposed to CM and reported its negative correlation to BC overall survival. In addition, ATF3 was demonstrated to inhibit the differentiation of 3T3-L1 adipocytes [72,73] and has been studied in obesity-related cancers, such as the colorectal one. However, ATF3’s specific role in obese BC patients remains unclear. Interestingly, ATF3 is linked to CEBP-β by CHAC1 [74], which has recently emerged as a hallmark of BC diagnosis and prognosis [75]. Moreover, it has been demonstrated that ATF3 genotypes/haplotypes cooperate with obesity to set the levels of C-reactive protein (CRP) [76], which is an acute-phase protein correlated with poor prognosis in various types of cancer [77] and obesity state [78]. A relationship between MMP polymorphisms and BC susceptibility [79,80,81] or obesity [82,83,84] has also been reported. Indeed, a recent study reported an obesity-specific association of MMP gene polymorphic loci with BC risk in postmenopausal women [85].

EPAS1, also known as hypoxia inducible factor 2 alpha (HIF-2α), represents a pivotal deregulated central hub of the DIN. Tumor hypoxia has been widely associated with malignancy, poor prognosis, and resistance to radiotherapy and chemotherapy [86]. Indeed, HIF-2α is frequently overexpressed in human cancers, and its canonical HIF-α–ARNT pathway activation is associated to the up-regulation of numerous cancer-relevant genes including VEGFA, PGK1 (Phosphoglycerate Kinase 1), and LOX (Lysyl Oxidase) for tumor angiogenesis, stemness, glycolysis, and metastasis, respectively [87,88,89,90]. A negative prognostic role of HIF-2α in patients suffering from different types of solid tumors was also found [91]. From a molecular point of view, EPAS1 was shown to regulate the expression of multiple glycolysis-related genes [92], and its interplay with SIPA1 is able to enhance aerobic metabolism along with BC metastasis [92]. In addition, EPAS1, after nuclear translocation, may activate genes involved in tumor angiogenesis, invasion, and metastasis [93]. Along with CEBP, EPAS1 converges in VEGFA, which is a key signaling component for angiogenesis processes [94]. Meta-analysis data from six randomized phase III trials in different cancers, including BC, suggested that variants in VEGF-A and EPAS1 are potential predictors of bevacizumab (anti-VEGF monoclonal antibody treatment) outcome [95]. It is also worth mentioning that obesity contributes to promoting anti-VEGF therapy resistance in BC [96].

The DIN central hub pool-like kinase 1 (PLK-1) plays key functions in multiple stages of the cell cycle, including the control of the G2/S checkpoint, centrosome maturation, spindle formation, chromosome segregation, DNA replication, cytokinesis, and meiosis [97,98], and its expression has been positively correlated with several cancers, including glioma [99], thyroid carcinoma [100], head and neck squamous cell carcinoma [101], melanoma [102], colorectal cancers [103], esophageal carcinoma [104], ovarian carcinoma [105], prostate cancer [106], and BC [107]. In vitro and in vivo studies proved that PLK1 is significantly correlated with metastasis, drug resistance, p53 mutation and stemness in BC cells [30,42,43,44,45,46,108]. In addition, PLK1 is a regulator of the cancer stem cell (CSC) phenotype whose inhibition prevents the stemness phenotype in cancer cells [42,46]. Interestingly, PLK1 correlates with the hormone-independent growth of ERα-positive BC cells, and its inhibition leads to a decrease in growth of long-term estrogen-deprived (LTED) BC cells [108]. Moreover, Jeon et al. found a high expression of PLK1 in Tamoxifen-resistant BC cells and demonstrated that PLK1 knockdown significantly decreases their proliferation, migration, and tumor growth [43]. In addition, Akdeli and colleagues identified rs27770 as a functional polymorphism that might affect cancer risk, progression and pharmacological resistance by modulating the secondary structure and stability of PLK1 mRNA [109].

In order to evaluate the potential clinical relevance of the DIN central hubs, retrospective analyses of the correlation among CEBP-β, PLK1, ATF3, EPAS1, and CEPB-zeta (DDIT3) levels and overall survival (OS) in patients with BC were conducted. Our results evidenced that only increased expression levels of CEBP-β or PLK1 can predict a significant poor prognosis in BC, and these proteins were then investigated in more detail.

### 4.2. Adipocyte-Derived CM and CEBP-β

The leucine-zipper TF family CEBPs, including CEBP-α, CEBP-β, CEBP-γ, CEBP-δ, CEBP-ε, and CEBP-ζ [33], play several roles in different physiological and pathological processes, such as energy metabolism, cell proliferation, and differentiation [34]. Named after the Roman god Janus, they have a double face, acting as tumor promoters and impacting proliferation and differentiation and drug/xenobiotic metabolism [110]. CEBP-β is a TF that is activated by phosphorylation and then translocated to the nucleus [36]. In addition, CEBPs participate in the regulation of pre-adipocyte differentiation. Inducers of differentiation lead to a rapid and transient expression, at both transcript and protein levels, of CEBP-β and CEBP-γ [33]. It is worth mentioning that cancer-associated adipocytes are pivotal inducers of inflammation and actively contribute to BC aggressiveness, invasion and metastasis [111]. Recently, the elevated expression of adipogenesis-related genes, such as CEBP-α, was correlated with a significantly worse OS in estrogen receptor (ER)-positive/HER2-negative BC patients and in triple-negative BC [112], whereas CEBP-β is dysregulated and appears to stimulate proliferation, colony formation, migration and invasion [113]. In agreement with these studies, Kurzejamska et al. showed CEBP-β as a predictor of OS in BC patients [114]. Regarding obesity/BC correlation, it was demonstrated that adipocyte-derived CM contains estradiol that activates ER-related genes involved in MCF-7 cell proliferation. This may reasonably explain why in CM-treated MCF-7 cells, we observed a strong up-regulation of C/EBP-β expression. Indeed, it has been well documented that C/EBP-β expression is rapidly induced in response to estradiol in epithelial and stroma compartments of the uterus [115]. The latter event was mediated by ER, since treatment with ER antagonists was able to suppress the expression of this transcription factor [115].

Three different CEBP-β isoforms exist: the liver-activating proteins LAP* and LAP, and liver inhibitory protein LIP [32,35]. While LIP is elevated in proliferative tissues and acts as an inhibitor of transcription, LAP1 and 2 are considered transcriptional activators, and their ratio is crucial for normal tissue growth and development. LIP expression is associated with mammary epithelial proliferation, and it was described in grade III and in estrogen receptor and progesterone receptor-negative BC [116]. The LIP/LAP ratio is also involved in BC invasion and migration and in the regulation of genes related to endothelial–mesenchymal transition (EMT) and to the extracellular matrix (ECM) [116]. LIP is an inducer of cancer metabolic reprogramming and is able to reduce levels of the let-7 microRNA, which is known as an oncosuppressor [117]. LAP expression is related to adipogenesis [118]. In this context, it is important to underline that the marked up-regulation of CEBP-β, which emerged from transcriptional analysis, was confirmed at the protein level. Indeed, immunoblotting analysis demonstrated that adipocyte-derived CM increased CEBP-β expression in both ERα-positive MCF-7 and -negative MDA-MB-231 breast cancer cells. Post-translational modifications (PTMs), mainly phosphorylations, play crucial roles in the regulation of C/EBP-β binding, transcriptional activity, protein–protein interactions and subcellular localization [32,119]. For instance, the transcriptional activity of C/EBP-β can be stimulated by the coexpression of oncogenic Ras through a mechanism that relies on the Thr235 phosphorylation on the latter [120]. Other important oncogenic signaling pathways overactivated in cancers, such as glycogen synthase kinase 3β (GSK3β) [36,121,122], Ca^2+^/calmodulin-dependent protein kinase [123], ribosomal S6 kinase [124,125], protein kinases A and C [126,127,128,129,130], and the cyclin-dependent kinase pathway CDK1–CDK2–CCNA2 (cyclinA) [36,131] can also mediate C/EBP-β phosphorylation. On the other hand, although other PTMs, including acetylation and methylation, may occur, they are mostly studied in adipogenesis control, and antibodies able to specifically detect the acetylated or methylated C/EBP-β proteoforms are not commercially available [32,119]. Interestingly, in addition to the rise in total protein levels, our results clearly showed an increase in the phosphorylation levels of LAP* and LIP in BC cells treated with CM. This is in line with several studies showing that obesity-related host signaling, including Insulin-like Growth Factor 1 (IGF-1) [132], Epidermal Growth Factor (EGF) [133,134] and IL pathways [135,136], may impact on CEBP-β expression and activity. On the other hand, this transcription factor has been found to regulate the expression of obesity-related bioactive molecules (i.e., IL-6, IGF-1, and leptin) [137,138,139], further suggesting the potential role of CEBP-β in mediating BC cell/adipocyte crosstalk.

In addition, the MetaCore enrichment analysis highlighted a consistent significant enrichment of genes involved in “AhR mediated regulation(_heart)”, and the AhR mediated regulation nets, DIN and SPN, are centered on CEBP-β. AhR is a ligand-dependent TF with a broad spectrum of expression that can be activated by various endogenous and exogenous molecules, including cellular metabolites, dietary and commensal compounds, and environmental pollutants. Its role in physiological and pathological states was widely described [140]. In particular, AhR is overexpressed and constitutively activated in advanced BC neoplasm and was shown to drive tumor progression [37]. The physiological functions of AhR during development appear to be the ancestral version of the adaptive xeno-sensor functions. The AhR repressor (AhRR) gene, which was first identified in mice as a target gene for AhR/Arnt trascription factors, acts as a negative regulator of AhR function by competing with AhR for dimerization with Arnt and binding to XRE. Lacking the Q-rich and PAS-B domains of AhR, AhRR is defective in both ligand binding and transcriptional activation, although AhRR shares a common ancestor with AhR. Experimental evidence addresses how AhRR might be suppressive on MCF-7 cell growth by altering the transcriptional and/or post-transcriptional regulation of ER- and cell cycle- related genes [141]. BC cells overexpressing AhRR exhibit a slow growth compared with parental cells, while cell cycle-related genes, such as E2F, cyclin D1, cyclin E1, and c-myc, were reduced [141]. Accordingly, AhRR appears to be down-regulated in CM-exposed MCF-7 cells in our experiments. AhR is also involved in the adipocyte differentiation process, where it negatively regulates adipocyte differentiation by repressing pRB phosphorylation [142]. The association of the AhR with pRB prevents the binding capacity of pRB to CEBP-β [143]. It is noteworthy that the non-canonical activation pathway of AhR involves its binding to the NF-kB subunit RelB, leading to the expression of pro-inflammatory genes, including CEBP-β [144]. Among the six factors directly crosslinked to CEBP-β in the AhR-regulated DIN, epiregulin (EREG) is the only CEBP-β interactor whose gene expression is up-regulated in our transcriptomics analysis. EREG is an EGF ligand implicated in angiogenesis, vascular remodeling, and cell proliferation [145], and it is positively regulated by CEPB-β. Moreover, AhR is able to increase epiregulin expression and gene transcription, which could play an important role in tumor promotion [145,146]. Additionally, exogenous EREG enhanced the survival of non-transformed breast epithelial cells in three-dimensional culture and in response to chemotherapeutic agents by regulating MMP-1 expression [147]. Up-regulated EREG levels were also described in human ductal carcinoma lesions compared to normal breast epithelium, and they predicted poor prognosis [147]. Interestingly, early-stage tumor cells are able to produce high levels of EREG as well as MMP-1 [147].

### 4.3. Adipocyte-Derived CM and PLK1

PLK1 belongs to a family of highly conserved serine/threonine protein kinases, along with PLK2, PLK3, and PLK4 members [148,149,150]. PLK1 activation requires the phosphorylation of a conserved threonine residue (Thr 210) in the PLK1 N-terminal kinase domain [151,152]. Bruinsma et al. demonstrated that although it is activated in the cytoplasm, PLK1 translocation to the nucleus is required for the phosphorylation of its target molecules [153]. PLK1 plays key roles in multiple stages of cell cycle, including the control of G2/S checkpoint, centrosome maturation, spindle formation, chromosome segregation, DNA replication, cytokinesis, and meiosis [97,98]. PLK1 is also required for genome stability preservation and DNA damage response [149,154]. Unsurprisingly, PLK1 is expressed in various types of malignancy and, despite some of them being described as a tumor suppressor (reviewed in [155,156]), its overexpression was widely correlated to poor prognosis in cancer, including BC [30,107,150,157,158,159,160,161,162,163,164]. In vitro and in vivo studies proved that PLK1 is significantly correlated with metastasis, drug resistance, p53 mutation and stemness in BC cells [30,42,43,44,45,46]. In addition, PLK1 is a regulator of the cancer stem cell (CSC) phenotype whose inhibition prevents the stemness phenotype in cancer cells [42,46]. Interestingly, PLK1 correlates with the hormone-independent growth of ERα-positive BC cells, and its inhibition leads to a decrease in growth of long-term estrogen-deprived (LTED) BC cells [108]. Moreover, Jeon et al. found a high expression of PLK1 in Tamoxifen-resistant BC cells and demonstrated that PLK1 knockdown significantly decreases their proliferation, migration, and tumor growth [43]. High PLK1 expression was also described in patient-derived xenografts (PDX) from bone metastasis compared to matched primary breast tumors [165]. It is noteworthy that Wierer et al. reported that PLK1 is able to mediate ER-regulated gene transcription in human BC cells [166], and this can be relevant since an enhanced production of estrogen in adipose tissue during obesity may occur [167]. Active PLK1-driven metastasis is also amplified by TGF-β signaling [168], whose activity is increased in advanced tumors as well as in obesity [169]. Interestingly, the obesity-related factors insulin [170] and leptin [171] have been reported to induce an increase in the expression of PLK1. In addition, high mRNA levels of PLK1 were described in MCF-7 cells co-cultured with visceral adipocyte stem cells (ASCs) along with induced EMT and enhanced proliferation, migration and invasion [172]. In line with these results, we found that exposure to adipocyte-derived CM leads to an increased abundance of total and phosphorylated isoform of PLK1 in MCF-7 and MDA-MB-231 BC cell lines. This knowledge may be important for the development of novel therapeutic approaches able to hamper the progression of obesity-mediated BCs. Indeed, more than 10 PLK1-specific inhibitors are commercially available, and some of them have been assessed in clinical trials for different types of cancers (reviewed in [173,174]). For instance, volasertib in combination with the chemotherapeutic agent cytarabine was demonstrated to induce an improved response rate and longer survival rates in comparison with cytarabine monotherapy in patients with advanced solid cancers [175].

In addition, the bioinformatic processing of transcriptomic results showed that CM-treated MCF-7 cells exhibited a significant enrichment of four factors annotated with the term “Cell cycle_role of APC in cell cycle regulation”, in which PLK1 is involved. The activity of anaphase-promoting complex (APC) is timed through the binding with the APC complex with two activating subunits: CDC20 during early mitosis until anaphases or CDH1 in late mitosis in G1 phases [176]. The activity of APC/CDC20 is regulated through inhibitory proteins or complexes which antagonize the function of CDC20: (i) the MAD2 or BUBR1, components of the spindle assembly checkpoint, and (ii) EMI1, an early mitotic inhibitor [177,178,179]. Previous findings reported that obese adipocytes may stimulate cell proliferation through a shift from the G1 phase to S and G2/M phases in T47D BC cells [180]. Our present data address how the CM from adipocytes may impact cell cycle regulation through an increased expression/activation of PLK1. To date, a lot of APC substrates involved in cell cycle regulation have been demonstrated to be overexpressed in several cancer types, including CDC20, the Aurora A and B kinases (AURKA and AURKB, respectively) and Forkhead box M1 (FOXM1) [181,182]. PLK1 after CDK1-dependent phosphorylation binds to the C-Terminal domain of FOXM1 and phosphorylates several residues of this TF, which in turn induces PLK1 overexpression by antagonizing its SUMOylation [164,183]. On the other hand, it has been reported how FOXM1 may up-regulate integrin β1 and potentiate FAK (focal adhesion kinase)-SRC signaling, which decelerates PLK1 degradation [184]. Thus, FOXM1 and PLK1 in their positive loop appear to be involved in cellular mitosis as well as in cellular invasiveness.

## 5. Conclusions

Obesity is a crucial risk factor in BC development and progression. Therefore, understanding the intricate connection linking obesity and BC is an unmet medical need. Here, we discussed the gene expression deregulation we observed in MCF-7 BC cells treated with adipocyte-derived conditioned medium (CM) to mimic the impact that obesity may have on BC development. Our data demonstrated a relevant gene modulation of several genes due to CM exposure, hence suggesting that obesity may affect a very large subset of transcripts in BC cells. Afterward, predictive functional analysis unraveled the presence of five highly relevant central hubs in the DIN, which are all involved in BC progression and spread. Particularly, it is important to note that CEBP-β and PLK1 are negatively correlated with BC prognosis in retrospective studies. Subsequent IB analysis demonstrated a CM-treatment dependent increase in the aforementioned factors also at the protein level. In conclusion, our findings suggest that the expression/activity of C/EBP-β and PLK1, factors already involved in BC aggressiveness as well as targets of different molecules dismissed by a dysfunctional adipose tissue, are exacerbated by adipocyte-derived CM. Hence, we can speculate that these factors, which negatively correlate with BC survival, may be relevant in mediating adipocyte/tumor cell crosstalk, and their roles deserve further investigation in the mechanisms involved in the obesity-induced BC development and progression.

## Figures and Tables

**Table 1:** Gene name, symbol, fold change and *p*-value of the five central hubs recovered in the transcriptomic analysis.

Gene Name	Gene Symbol	FC (CM-Treated vs. Control)	padj
CCAAT enhancer binding protein beta	*CEBPB*	6.98	6.96 × 10^−288^
Polo-Like Kinase 1	*PLK1*	4.47	2.00 × 10^−20^
Activating Transcription Factor 3	*ATF3*	5.53	4.14 × 10^−39^
Endothelial PAS Domain Protein 1	*EPAS1*	−4.54	4.35 × 10^−106^
Damage-inducible Transcript 3 Protein	*DDIT3*	12.51	0

FC: fold change, padj: adjusted *p*-value.

## Data Availability

All the data are available in a public, open-access repository.

## Supplementary Materials

The following supporting information can be downloaded at: https://www.mdpi.com/article/10.3390/nu15132839/s1, Figure S1: (**a**) Representative phase contrast (*upper panel*) and oil red O staining (*lower panel*) images of 3T3-L1 cells at the preadipocyte and adipocyte (A) stages (40×). (**b**) Quantitative results of oil red O staining images of 3T3-L1 and 3T3-L1 A cells. (**c**) Real-time RT-PCR for different adipocyte markers. Mean ± SEM. * *p* < 0.05; ** *p* < 0.005; *** *p* < 0.0005. Figure S2: DIN in central-hub centred trace-mode and mark-up view of shortest path interconnections among central hubs. FDR ≥ 0.01 and fold change −2 ≥ log2FC ≥ 2. CEBPβ: CCAAT/enhancer binding protein beta; ATF-3: cyclic AMP-dependent transcription factor: ATF-3; CEBPζ: CCAAT/enhancer-binding protein zeta; EPAS1: endothelial PAS domain-containing protein 1 PLK1: polo-like kinase 1. Figure S3: Kaplan-Meyer survival analysis correlating the levels Of VEGFA (Vascular Endothelial Growth Factor A), MMP1 (Matrix Metallopeptidase 1), or CHAC1 (ChaC Glutathione Specific Gamma-Glutamylcyclotransferase 1) and overall survival in breast cancer patients. Kaplan-Meier survival graph and hazard ratio with 95% confidence intervals and logrank *p* value are shown. Figure S4: SPN built by processing the 28 CM-deregulated-genes annotated by the significantly enriched CFDR 1.07 × 10^−12^) term “AhR mediated regulation (heart)” in the MetaCore “Drug and Xenobiotic Metabolism Enzymes ontology. All the experimental factors entered into the net and CEBP: CCAAT/enhancer binding protein beta, EPAS1: endothelial PAS domain-containing protein 1 VEGFR-2:Vascular Endothelial Growth Factor Receptor-2, SLC3A2: solute carrier family 3 member 2 and amphiregulin resulted the central hubs. Table S1: Oligonucleotide primers for Real Time PCR assay; Table S2: CM-deregulated genes (FDR ≥ 0.01 and fold change −2 ≥ log2FC ≥ 2); Table S3: Twenty-eight of the 364 deregulated genes (FDR ≥ 0.01 and fold change −2 ≥ log2FC ≥ 2) between CM untreated (control) and CM treated (CMT) MCF-7 BC cells.
