# Supplementary material for: CEBP-β and PLK1 as Potential Mediators of the Breast Cancer/Obesity Crosstalk: In Vitro and In Silico Analyses
Source: Nutrients. 2023 Jun 22;15(13):2839. doi: 10.3390/nu15132839 (PMC10343266; doi:10.3390/nu15132839)
Supplement: Supplementary file 1 [file nutrients-15-02839-s001.zip › nutrients-2438000-supplementary/Supplementary Table S1.pdf]

Supplementary Table S1. Oligonucleotide primers for Real Time PCR assay.

Gene Name	Gene Symbol	Species		Primer Sequences
Adiponectin, C1Q and Collagen Domain Containing	*ADIPOQ*	Murine	Forward<br>Reverse	5′- TGACGACACCAAAAGGGCTC-3′<br>5′ -CACAAGTTCCCTTGGGTGGA-3′
Actin Alpha 2, Smooth Muscle	*ACTA2*	Murine	Forward<br>Reverse	5′- AGCCATCTTTCATTGGGAT-3′<br>5′- CATGGTGGTACCCCCTGACA-3′
Fatty Acid Binding Protein 4	*FABP4*	Murine	Forward<br>Reverse	5′- ATGAAATCACCGCAGACGAC-3′<br>5′- TCATAACACATTCCACCACC-3′
Matrix Metallopeptidase 9	*MMP9*	Murine	Forward<br>Reverse	5′- AAGGCAGCGTTAGCCAGAA-3′<br>5′ -GCGGTACAAGTATGCCTCT-3′
Ob	*Ob*	Murine	Forward<br>Reverse	5′- ACTTCATTCCTGGGCTTCAC-3′<br>5′ -GATTCTCCAGGTCATTGGCT-3′
Peroxisome Proliferator-Activated Receptor Gamma	*PPARG*	Murine	Forward<br>Reverse	5′- AGGAGCCTGTGAGACCAACA-3′<br>5′- CACCGCTTCTTTCAAATCTT-3′
