# Supplementary material for: CEBP-β and PLK1 as Potential Mediators of the Breast Cancer/Obesity Crosstalk: In Vitro and In Silico Analyses
Source: Nutrients. 2023 Jun 22;15(13):2839. doi: 10.3390/nu15132839 (PMC10343266; doi:10.3390/nu15132839)
Supplement: Supplementary file 1 [file nutrients-15-02839-s001.zip › nutrients-2438000-supplementary/Supplementary Table S2.pdf]

Supplementary Table S2. CM-deregulated genes (FDR ≥ 0.01 and fold change -2 ≥ log2FC ≥ 2).

Gene	baseMean	log2FoldChange	FC	FoldChange	lfcSE	pvalue	padj
TXNIP	4325,08	-4,40	0,05	-21,15	0,07	0,00E+00	0,00E+00
IGFBP5	4094,24	-3,09	0,12	-8,54	0,07	0,00E+00	0,00E+00
BCAS1	2825,98	-2,96	0,13	-7,80	0,06	0,00E+00	0,00E+00
PMP22	2202,03	-2,66	0,16	-6,32	0,07	0,00E+00	0,00E+00
XPOT	7965,76	2,04	4,12	4,12	0,04	0,00E+00	0,00E+00
MARS1	3538,39	2,08	4,22	4,22	0,06	0,00E+00	0,00E+00
AARS1	12453,74	2,08	4,23	4,23	0,05	0,00E+00	0,00E+00
GARS1	3271,24	2,17	4,49	4,49	0,06	0,00E+00	0,00E+00
VEGFA	2333,20	2,32	5,00	5,00	0,06	0,00E+00	0,00E+00
WARS1	3908,15	2,45	5,48	5,48	0,05	0,00E+00	0,00E+00
HSPA5	18008,41	2,72	6,59	6,59	0,05	0,00E+00	0,00E+00
DDIT3	1133,35	3,65	12,51	12,51	0,09	0,00E+00	0,00E+00
ALDH1L2	1104,74	4,23	18,77	18,77	0,10	0,00E+00	0,00E+00
SLC7A11	1121,19	4,36	20,60	20,60	0,10	0,00E+00	0,00E+00
EGR3	1511,01	4,89	29,57	29,57	0,10	0,00E+00	0,00E+00
CEBPB	1161,28	2,80	6,98	6,98	0,08	8,39E-291	6,96E-288
SLC3A2	18319,91	2,31	4,96	4,96	0,07	3,73E-260	2,52E-257
PABPC1L	2480,87	2,53	5,76	5,76	0,08	1,12E-245	6,83E-243
SOCS2	1066,75	-2,47	0,18	-5,52	0,08	2,11E-230	1,24E-227
ERRFI1	983,95	2,84	7,15	7,15	0,09	2,48E-228	1,42E-225
DOK7	1093,90	2,18	4,55	4,55	0,07	1,97E-188	8,98E-186
ADM2	491,01	3,81	13,98	13,98	0,13	3,57E-185	1,59E-182
AC055854.1	483,38	3,58	11,96	11,96	0,13	2,67E-179	1,11E-176
AQP3	1207,57	-2,07	0,24	-4,19	0,07	1,80E-172	7,00E-170
CLMN	1350,11	-2,25	0,21	-4,75	0,08	1,93E-168	7,18E-166
CEBPG	988,52	2,26	4,78	4,78	0,08	4,10E-168	1,50E-165
VTCN1	458,82	-3,90	0,07	-14,95	0,14	1,81E-166	6,34E-164
NUPR1	964,79	2,20	4,60	4,60	0,08	2,25E-163	7,75E-161
HMGCS2	360,07	-4,31	0,05	-19,83	0,16	6,51E-152	1,92E-149
MANF	760,63	2,55	5,84	5,84	0,10	2,70E-150	7,70E-148
LRATD1	756,83	-2,25	0,21	-4,76	0,09	4,65E-148	1,29E-145
CDC42EP1	862,02	2,01	4,02	4,02	0,08	1,87E-144	5,03E-142
PSCA	315,34	-3,98	0,06	-15,80	0,16	5,36E-133	1,29E-130
CRELD2	760,76	2,03	4,09	4,09	0,08	4,03E-131	9,07E-129
GRIK3	351,92	3,31	9,90	9,90	0,14	6,25E-129	1,33E-126
LDLRAD4	881,36	-2,02	0,25	-4,04	0,09	7,83E-120	1,50E-117
IL1R1	378,88	-2,77	0,15	-6,80	0,12	1,63E-117	3,03E-115
PHGDH	3499,65	2,84	7,14	7,14	0,13	1,77E-114	3,17E-112
AREG	263,63	4,18	18,07	18,07	0,19	1,16E-108	2,04E-106
EPAS1	502,12	-2,18	0,22	-4,54	0,10	2,50E-108	4,35E-106
SDF2L1	462,12	2,44	5,43	5,43	0,11	4,84E-108	8,33E-106
CLEC3A	297,80	-3,04	0,12	-8,24	0,14	1,52E-106	2,57E-104
AKNA	596,00	2,17	4,50	4,50	0,10	4,14E-102	6,34E-100
MAP1B	353,27	2,86	7,24	7,24	0,14	1,02E-98	1,48E-96
ADORA1	359,52	2,54	5,81	5,81	0,12	1,91E-97	2,74E-95

IL17REL	242,55	-3,75	0,07	-13,42	0,18	4,77E-97	6,75E-95
SMOX	475,97	2,25	4,77	4,77	0,11	1,12E-95	1,54E-93
CHAC1	242,33	4,85	28,84	28,84	0,23	1,89E-95	2,58E-93
CDCA5	527,22	2,26	4,81	4,81	0,11	1,21E-93	1,59E-91
PDLIM3	344,12	2,46	5,51	5,51	0,12	4,93E-93	6,42E-91
SLITRK6	425,11	2,20	4,59	4,59	0,11	1,04E-91	1,30E-89
CSTA	230,51	-3,31	0,10	-9,94	0,16	9,59E-91	1,18E-88
TRGC1	697,21	-2,02	0,25	-4,06	0,10	1,03E-90	1,25E-88
SYNE3	312,36	2,67	6,39	6,39	0,14	5,24E-88	6,09E-86
RIPOR3	365,49	-2,37	0,19	-5,17	0,13	9,48E-81	9,78E-79
SMPD3	483,62	-2,19	0,22	-4,55	0,12	6,47E-79	6,53E-77
HSPA13	389,79	2,04	4,11	4,11	0,11	1,08E-78	1,09E-76
ALDH1A3	324,91	-2,58	0,17	-5,99	0,14	8,16E-78	8,09E-76
BEX2	251,95	2,60	6,07	6,07	0,14	2,17E-76	2,11E-74
NIBAN1	248,86	6,06	66,84	66,84	0,33	1,23E-75	1,15E-73
PTPRN2	258,32	-2,69	0,15	-6,47	0,15	2,57E-75	2,40E-73
SLC24A3	242,02	-2,69	0,16	-6,45	0,15	1,56E-73	1,41E-71
BMF	258,00	-2,57	0,17	-5,93	0,15	3,90E-71	3,37E-69
FICD	298,69	2,18	4,52	4,52	0,12	2,18E-70	1,88E-68
CLGN	168,80	4,70	25,98	25,98	0,27	4,68E-68	3,80E-66
ATP8A1	432,69	-2,10	0,23	-4,28	0,12	5,46E-68	4,39E-66
CEP55	320,91	2,07	4,19	4,19	0,12	7,68E-67	6,07E-65
KCNE4	155,80	3,98	15,82	15,82	0,24	2,89E-64	2,17E-62
LIMCH1	348,86	-2,33	0,20	-5,04	0,14	4,22E-64	3,16E-62
MYBL1	236,35	2,45	5,45	5,45	0,15	1,04E-62	7,53E-61
RND1	228,89	-2,52	0,17	-5,75	0,15	2,30E-61	1,62E-59
HMOX1	299,78	2,31	4,96	4,96	0,14	4,32E-61	2,99E-59
TNFSF10	184,96	-2,81	0,14	-7,00	0,17	1,20E-59	8,05E-58
UGT3A2	257,87	-2,19	0,22	-4,56	0,14	4,61E-59	3,06E-57
SLC7A5	59363,04	2,10	4,28	4,28	0,13	4,75E-59	3,14E-57
NAV2	330,36	2,22	4,67	4,67	0,14	4,93E-59	3,25E-57
IQGAP2	187,02	-3,00	0,12	-8,03	0,19	5,65E-58	3,64E-56
ULBP1	156,99	3,02	8,09	8,09	0,19	1,45E-56	9,00E-55
GRM4	214,03	-2,27	0,21	-4,82	0,14	2,96E-56	1,82E-54
CDC6	248,25	2,19	4,57	4,57	0,14	4,62E-56	2,82E-54
TUBE1	239,24	2,18	4,55	4,55	0,14	4,88E-56	2,97E-54
CYBRD1	163,08	-3,28	0,10	-9,70	0,21	1,43E-55	8,53E-54
CHST1	128,11	-4,37	0,05	-20,75	0,28	9,93E-55	5,72E-53
PIF1	169,22	2,63	6,20	6,20	0,17	2,49E-52	1,37E-50
AC022034.1	211,28	-2,25	0,21	-4,74	0,15	1,78E-51	9,56E-50
LIPH	211,52	-2,28	0,21	-4,86	0,15	7,44E-51	3,94E-49
KLHDC7B	123,66	4,71	26,11	26,11	0,32	2,37E-50	1,22E-48
CDKN3	282,65	2,09	4,27	4,27	0,14	3,28E-50	1,67E-48
PPP1R3C	171,78	-2,42	0,19	-5,36	0,17	2,19E-49	1,10E-47
CCN5	127,46	3,51	11,42	11,42	0,24	2,51E-49	1,25E-47
SOCS2-AS1	212,20	-2,16	0,22	-4,46	0,15	8,97E-48	4,33E-46
ST6GAL1	214,73	-2,14	0,23	-4,42	0,15	1,35E-47	6,51E-46
F7	130,48	3,02	8,11	8,11	0,21	2,22E-47	1,06E-45

CDCA3	282,71		2,06	4,18	4,18	0,15	4,50E-46	2,09E-44
DEPP1	109,97		-4,41	0,05	-21,20	0,31	1,45E-45	6,69E-44
LOXL4	114,74		3,20	9,16	9,16	0,23	2,37E-44	1,05E-42
KIF2C	225,70		2,09	4,26	4,26	0,15	8,63E-44	3,75E-42
NDC80	198,70		2,33	5,04	5,04	0,17	2,22E-43	9,51E-42
ATF3	143,18		2,47	5,53	5,53	0,19	1,04E-40	4,14E-39
CENPA	203,01		2,07	4,20	4,20	0,16	1,59E-40	6,32E-39
SKA3	176,25		2,05	4,13	4,13	0,16	1,33E-39	5,14E-38
MME	91,70		-3,95	0,06	-15,44	0,30	1,62E-39	6,26E-38
BEST1	185,35		7,19	145,80	145,80	0,55	7,08E-39	2,66E-37
GABBR2	132,84		-2,51	0,18	-5,70	0,20	5,05E-36	1,78E-34
SGO1	134,39		2,41	5,30	5,30	0,20	1,77E-35	6,13E-34
SALL2	154,09		-2,03	0,25	-4,08	0,17	3,25E-35	1,10E-33
CKAP2L	158,96		2,04	4,10	4,10	0,17	3,75E-35	1,27E-33
DRAIC	88,37		-3,07	0,12	-8,42	0,26	3,78E-34	1,24E-32
DAB2	109,25		-2,45	0,18	-5,48	0,21	6,41E-34	2,08E-32
PKMYT1	922,28		2,26	4,80	4,80	0,19	8,86E-34	2,86E-32
CYP1A1	146,91		-2,30	0,20	-4,93	0,19	2,23E-33	7,07E-32
COL28A1	86,18		-2,92	0,13	-7,55	0,25	4,83E-33	1,49E-31
TSPAN12	116,88		-2,20	0,22	-4,60	0,19	4,17E-31	1,21E-29
HLA-DRB1	123,83		-2,05	0,24	-4,15	0,18	9,44E-31	2,69E-29
CMTM8	127,88		2,04	4,11	4,11	0,18	3,25E-30	9,10E-29
BHLHA15	68,01		4,11	17,25	17,25	0,36	3,56E-30	9,97E-29
PALM2AKAP2	101,27		-2,33	0,20	-5,05	0,21	4,32E-30	1,20E-28
GADD45A	119,84		2,09	4,25	4,25	0,19	2,03E-29	5,49E-28
ASNSP1	66,16		4,56	23,63	23,63	0,41	8,90E-29	2,35E-27
KCNJ8	65,55		-3,38	0,10	-10,41	0,31	2,50E-28	6,49E-27
TNFSF15	116,32		-2,04	0,24	-4,12	0,19	5,54E-28	1,42E-26
C2orf72	57,87		-4,19	0,05	-18,22	0,39	2,98E-27	7,27E-26
UBE2C	646,07		2,39	5,25	5,25	0,23	8,42E-27	1,99E-25
PHEX	127,15		-2,00	0,25	-4,01	0,19	1,43E-26	3,36E-25
LIF	122,86		2,02	4,06	4,06	0,19	1,48E-26	3,46E-25
MSMB	101,01		-2,16	0,22	-4,47	0,21	1,51E-26	3,53E-25
PIP5KL1	98,55		2,35	5,11	5,11	0,23	3,14E-26	7,27E-25
CCNA1	58,47		3,62	12,28	12,28	0,35	8,27E-26	1,87E-24
PLEKHD1	65,33		-3,06	0,12	-8,32	0,30	1,16E-25	2,60E-24
ADAM12	54,39		-3,73	0,08	-13,24	0,36	1,28E-25	2,85E-24
AMTN	71,22		-2,67	0,16	-6,37	0,26	1,78E-25	3,93E-24
SLC26A1	100,17		2,09	4,25	4,25	0,21	5,31E-24	1,08E-22
MSTO2P	83,65		2,36	5,13	5,13	0,24	5,35E-24	1,09E-22
KRT89P	76,99		-2,41	0,19	-5,31	0,25	2,34E-23	4,64E-22
AC103760.1	72,42		-2,56	0,17	-5,90	0,27	4,04E-23	7,94E-22
RASGRP3	53,61		-3,21	0,11	-9,22	0,33	4,17E-23	8,18E-22
VLDLR-AS1	51,45		3,57	11,91	11,91	0,37	1,75E-22	3,32E-21
RDH12	64,97		5,84	57,33	57,33	0,60	5,05E-22	9,26E-21
PLK1	840,42		2,16	4,47	4,47	0,23	1,11E-21	2,00E-20
IL20	91,02		2,02	4,06	4,06	0,22	3,05E-21	5,38E-20
COL2A1	53,20		-2,91	0,13	-7,50	0,32	3,63E-21	6,38E-20

SERHL2	64,15	-2,53	0,17	-5,78	0,28	4,21E-21	7,34E-20
MOV10L1	61,58	2,48	5,58	5,58	0,28	2,41E-20	4,02E-19
MEG3	62,61	-2,36	0,19	-5,15	0,26	2,81E-20	4,67E-19
FGG	43,23	3,71	13,08	13,08	0,41	6,21E-20	1,01E-18
EML2-AS1	69,18	2,25	4,76	4,76	0,25	1,15E-19	1,84E-18
ASNS	66,83	2,30	4,93	4,93	0,26	1,22E-19	1,94E-18
KCNK3	86,53	-2,23	0,21	-4,70	0,25	1,91E-19	3,00E-18
SERPINA11	40,31	-4,23	0,05	-18,75	0,49	1,25E-18	1,87E-17
PRR11	1015,19	2,08	4,24	4,24	0,25	2,63E-18	3,85E-17
RASL11A	46,18	-2,81	0,14	-7,03	0,34	5,95E-18	8,47E-17
ANK1	39,89	-3,17	0,11	-8,98	0,38	8,29E-18	1,17E-16
COL3A1	63,21	-2,14	0,23	-4,42	0,26	1,57E-17	2,16E-16
SPATA41	40,48	2,94	7,69	7,69	0,37	1,08E-16	1,41E-15
VAT1L	55,97	-2,23	0,21	-4,70	0,28	1,55E-16	2,00E-15
MAB21L4	54,41	-2,62	0,16	-6,14	0,33	1,70E-16	2,18E-15
BPIFA4P	36,01	-3,44	0,09	-10,88	0,43	1,92E-16	2,45E-15
GAL	61,67	2,25	4,76	4,76	0,28	2,22E-16	2,82E-15
DMGDH	34,60	4,01	16,08	16,08	0,50	2,75E-16	3,44E-15
AP003119.1	51,88	2,36	5,13	5,13	0,30	3,71E-16	4,62E-15
STON1	50,21	-2,40	0,19	-5,26	0,31	5,97E-16	7,33E-15
TUBA3D	33,25	4,06	16,67	16,67	0,52	1,60E-15	1,92E-14
NDUFA4L2	38,50	3,03	8,15	8,15	0,39	1,66E-15	1,98E-14
DTX4	57,30	-2,11	0,23	-4,30	0,28	2,67E-15	3,16E-14
DEPDC4	40,18	2,83	7,13	7,13	0,37	2,84E-15	3,35E-14
AC010327.7	35,07	4,89	29,64	29,64	0,63	3,06E-15	3,61E-14
IL24	32,40	4,36	20,59	20,59	0,56	3,61E-15	4,23E-14
RASIP1	32,31	3,51	11,42	11,42	0,47	8,00E-15	9,17E-14
RFPL1S	62,74	2,16	4,46	4,46	0,29	8,29E-15	9,48E-14
GRHL3	44,76	-2,40	0,19	-5,29	0,33	2,37E-14	2,63E-13
BPIFB1	26,83	-4,00	0,06	-15,96	0,56	1,29E-13	1,34E-12
CDC20	541,48	2,18	4,53	4,53	0,31	2,35E-13	2,38E-12
JAM2	35,32	-2,65	0,16	-6,27	0,38	3,24E-13	3,24E-12
AL158847.1	27,55	-3,27	0,10	-9,67	0,47	3,56E-13	3,55E-12
DIRC3	35,33	-2,59	0,17	-6,02	0,37	3,72E-13	3,70E-12
LRP2	34,67	-2,78	0,15	-6,85	0,40	4,65E-13	4,58E-12
AC093904.4	45,21	-2,13	0,23	-4,39	0,31	7,52E-13	7,31E-12
P2RX2	27,07	-3,04	0,12	-8,22	0,45	1,88E-12	1,77E-11
AL732437.3	31,80	-2,58	0,17	-5,99	0,39	2,02E-12	1,90E-11
ACHE	29,65	-3,02	0,12	-8,09	0,45	2,53E-12	2,35E-11
MYZAP	31,74	-2,52	0,17	-5,72	0,38	3,78E-12	3,49E-11
GCOM1	36,27	-2,28	0,21	-4,85	0,35	8,15E-12	7,28E-11
ENPP2	24,72	4,10	17,19	17,19	0,62	8,55E-12	7,64E-11
AC270286.1	36,64	2,29	4,91	4,91	0,35	1,08E-11	9,52E-11
CP	55,42	-2,06	0,24	-4,16	0,32	1,13E-11	9,99E-11
CRISP3	26,81	-2,86	0,14	-7,25	0,45	1,47E-11	1,28E-10
SLC2A14	23,87	4,28	19,44	19,44	0,65	1,51E-11	1,31E-10
SVOPL	32,88	-2,34	0,20	-5,07	0,37	1,56E-11	1,36E-10
LINC00930	31,11	-2,49	0,18	-5,62	0,39	1,89E-11	1,64E-10

CACNG6	22,32	-3,71	0,08	-13,05	0,58	2,61E-11	2,22E-10
FAM72D	32,59	2,49	5,61	5,61	0,39	2,69E-11	2,29E-10
ERP27	26,36	-2,99	0,13	-7,96	0,48	3,27E-11	2,75E-10
DYNLRB2	40,66	-2,09	0,24	-4,25	0,33	3,58E-11	3,00E-10
CRYM	23,55	-3,22	0,11	-9,33	0,51	4,15E-11	3,46E-10
PGM5	38,57	-2,15	0,23	-4,44	0,35	5,00E-11	4,13E-10
FES	21,67	3,71	13,12	13,12	0,59	7,20E-11	5,88E-10
IGDCC3	25,57	-2,85	0,14	-7,22	0,46	7,40E-11	6,03E-10
COLGALT2	42,82	2,01	4,04	4,04	0,33	9,45E-11	7,64E-10
AC099521.2	22,40	-2,82	0,14	-7,06	0,49	7,33E-10	5,43E-09
AC009093.4	18,05	8,17	288,09	288,09	2,65	7,76E-10	5,73E-09
ANK2	24,94	2,64	6,22	6,22	0,45	7,81E-10	5,76E-09
AC098935.1	25,79	2,69	6,46	6,46	0,47	8,46E-10	6,22E-09
LINC02489	31,06	-2,10	0,23	-4,29	0,37	1,45E-09	1,05E-08
MUC3A	33,16	-2,01	0,25	-4,03	0,36	2,59E-09	1,82E-08
ALPL	31,05	-2,04	0,24	-4,11	0,37	2,59E-09	1,82E-08
KRT4	17,75	-3,49	0,09	-11,25	0,62	2,93E-09	2,04E-08
NBPF13P	20,66	-2,78	0,15	-6,86	0,50	3,19E-09	2,22E-08
KIF21B	19,45	3,16	8,95	8,95	0,57	3,25E-09	2,26E-08
AC116407.1	25,86	-2,27	0,21	-4,83	0,41	3,43E-09	2,37E-08
ASCL1	29,61	2,12	4,36	4,36	0,39	3,44E-09	2,38E-08
WDFY4	25,05	2,36	5,13	5,13	0,43	3,45E-09	2,38E-08
ZMIZ1-AS1	18,99	3,17	8,99	8,99	0,57	3,63E-09	2,50E-08
MIR205HG	28,45	-2,25	0,21	-4,77	0,41	3,70E-09	2,54E-08
PRRT1B	20,85	-2,68	0,16	-6,43	0,49	4,09E-09	2,80E-08
AC010998.3	19,61	-2,90	0,13	-7,47	0,53	5,25E-09	3,55E-08
MAP2K6	18,71	-3,08	0,12	-8,45	0,56	5,40E-09	3,65E-08
SERPINA3	20,05	2,83	7,13	7,13	0,52	5,59E-09	3,77E-08
C5AR1	23,48	2,33	5,02	5,02	0,44	1,37E-08	8,88E-08
RSPO1	21,52	2,49	5,63	5,63	0,48	1,76E-08	1,12E-07
PROB1	25,55	2,22	4,66	4,66	0,43	1,94E-08	1,23E-07
LMO3	19,48	-2,65	0,16	-6,30	0,52	2,50E-08	1,56E-07
ITGA9	15,36	-3,43	0,09	-10,81	0,66	2,83E-08	1,75E-07
AL136090.2	20,32	-2,52	0,17	-5,74	0,49	2,97E-08	1,83E-07
PIP	17,90	-2,73	0,15	-6,65	0,54	3,82E-08	2,34E-07
LINC01762	18,28	-2,65	0,16	-6,27	0,52	3,90E-08	2,38E-07
KCNJ3	18,30	-2,67	0,16	-6,35	0,53	3,96E-08	2,41E-07
SLPI	24,09	2,33	5,03	5,03	0,46	4,03E-08	2,46E-07
MSLNL	23,28	2,31	4,97	4,97	0,46	4,14E-08	2,52E-07
RTP4	26,67	-2,07	0,24	-4,19	0,41	4,25E-08	2,58E-07
NRK	20,67	-2,39	0,19	-5,23	0,47	4,29E-08	2,60E-07
GSTM5	16,10	-3,00	0,13	-7,98	0,59	5,06E-08	3,04E-07
IZUMO1	17,55	2,89	7,41	7,41	0,57	5,69E-08	3,39E-07
SPTLC3	20,78	-2,36	0,20	-5,12	0,48	6,76E-08	4,01E-07
ITGB6	25,69	-2,09	0,24	-4,24	0,43	8,27E-08	4,87E-07
WNT3A	16,08	-3,29	0,10	-9,76	0,66	8,56E-08	5,03E-07
CDC20P1	19,44	2,52	5,73	5,73	0,52	9,77E-08	5,71E-07
RGCC	23,37	-2,08	0,24	-4,23	0,43	1,03E-07	6,02E-07

SYPL1P2	24,45	2,03	4,08	4,08	0,42	1,06E-07	6,17E-07
ZNF536	16,30	-2,86	0,14	-7,28	0,58	1,06E-07	6,19E-07
AL357033.4	19,31	-2,41	0,19	-5,32	0,51	1,52E-07	8,71E-07
SILC1	13,83	-3,27	0,10	-9,63	0,68	1,92E-07	1,08E-06
KDR	14,34	-3,13	0,11	-8,74	0,65	1,96E-07	1,11E-06
PDE11A	22,12	-2,38	0,19	-5,20	0,51	2,42E-07	1,35E-06
AC126696.3	15,55	5,87	58,36	58,36	1,30	2,71E-07	1,50E-06
DRD2	21,44	-2,10	0,23	-4,27	0,45	3,13E-07	1,72E-06
AC139887.1	16,68	2,64	6,22	6,22	0,57	3,20E-07	1,76E-06
CDC42EP3	22,42	2,03	4,07	4,07	0,44	3,88E-07	2,11E-06
NCALD	18,91	-2,27	0,21	-4,83	0,50	3,99E-07	2,16E-06
FUT3	16,00	2,66	6,31	6,31	0,58	4,31E-07	2,32E-06
AC022239.1	12,99	3,55	11,72	11,72	0,75	4,74E-07	2,54E-06
NEIL3	52,56	2,41	5,31	5,31	0,55	6,33E-07	3,33E-06
SEMA3G	20,66	2,07	4,20	4,20	0,46	6,60E-07	3,47E-06
AC093904.2	16,05	-2,44	0,18	-5,42	0,55	8,77E-07	4,54E-06
TUBA3E	12,70	4,22	18,62	18,62	0,90	8,99E-07	4,64E-06
INHBE	13,08	5,57	47,61	47,61	1,31	9,34E-07	4,81E-06
LINC00471	17,90	2,36	5,12	5,12	0,54	1,07E-06	5,49E-06
LINC02343	14,89	-2,55	0,17	-5,87	0,58	1,17E-06	5,98E-06
AC126696.2	12,63	3,21	9,23	9,23	0,73	1,32E-06	6,72E-06
KCNJ13	6,97	-6,56	0,01	-94,36	2,57	1,33E-06	6,77E-06
LINC02233	11,35	-3,73	0,08	-13,26	0,83	1,37E-06	6,95E-06
AC046168.1	14,28	2,62	6,16	6,16	0,61	1,62E-06	8,14E-06
PLEKHG1	18,33	2,14	4,41	4,41	0,50	1,63E-06	8,17E-06
MARK1	17,21	2,23	4,71	4,71	0,52	1,72E-06	8,58E-06
APELA	11,16	-3,71	0,08	-13,12	0,83	1,72E-06	8,59E-06
THEM5	7,22	6,45	87,27	87,27	2,52	1,73E-06	8,64E-06
TRPV3	11,34	4,01	16,12	16,12	0,91	2,72E-06	1,32E-05
HOXA11	16,41	2,21	4,61	4,61	0,53	2,94E-06	1,42E-05
NRP2	16,89	-2,10	0,23	-4,28	0,51	3,36E-06	1,62E-05
AHRR	16,76	-2,08	0,24	-4,24	0,52	4,65E-06	2,19E-05
LINC01160	10,75	3,52	11,48	11,48	0,84	4,76E-06	2,24E-05
CPNE7	16,79	-2,08	0,24	-4,23	0,52	4,82E-06	2,26E-05
SH2D3C	15,76	2,31	4,97	4,97	0,59	5,82E-06	2,71E-05
IL21R	5,93	6,07	67,11	67,11	2,49	6,53E-06	3,02E-05
SLC7A7	5,89	6,05	66,12	66,12	2,49	6,85E-06	3,15E-05
CEACAM7	15,79	-2,05	0,24	-4,15	0,52	7,33E-06	3,36E-05
KRT16	10,84	3,17	9,01	9,01	0,79	7,46E-06	3,41E-05
SEMA5A	10,07	-3,52	0,09	-11,49	0,87	7,63E-06	3,48E-05
AC131009.2	16,22	2,10	4,30	4,30	0,54	7,94E-06	3,61E-05
AC034213.1	10,04	3,34	10,12	10,12	0,85	1,26E-05	5,60E-05
TRPV2	12,16	2,49	5,63	5,63	0,66	1,41E-05	6,22E-05
AC026202.2	11,31	2,69	6,47	6,47	0,72	1,67E-05	7,31E-05
KIAA0319	12,32	2,33	5,01	5,01	0,64	2,24E-05	9,61E-05
ADGRA2	14,31	2,08	4,23	4,23	0,58	2,72E-05	1,15E-04
LUCAT1	7,93	4,74	26,70	26,70	1,35	2,81E-05	1,19E-04
CDH5	9,84	-2,88	0,14	-7,34	0,80	3,03E-05	1,27E-04

VWA3B	10,42	2,58	5,99	5,99	0,73	3,43E-05	1,42E-04
CYP4F3	12,44	2,19	4,56	4,56	0,62	3,48E-05	1,44E-04
ARHGAP9	10,42	2,59	6,01	6,01	0,74	4,04E-05	1,66E-04
CYP2E1	11,09	-2,43	0,19	-5,37	0,70	4,20E-05	1,72E-04
ZBP1	13,35	-2,03	0,24	-4,09	0,59	4,45E-05	1,82E-04
COLEC12	10,02	-2,78	0,15	-6,87	0,81	4,82E-05	1,96E-04
NEFH	13,59	2,06	4,16	4,16	0,61	5,60E-05	2,25E-04
CADPS	8,45	3,04	8,25	8,25	0,88	6,66E-05	2,65E-04
PLSCR4	8,22	-2,78	0,15	-6,86	0,84	9,23E-05	3,60E-04
HMX3	6,44	4,35	20,34	20,34	1,38	1,06E-04	4,10E-04
IL1RN	12,32	2,09	4,26	4,26	0,67	1,20E-04	4,59E-04
OTOF	9,89	2,27	4,84	4,84	0,71	1,21E-04	4,63E-04
SNORD3A	11,12	-2,22	0,22	-4,65	0,71	1,22E-04	4,65E-04
AC116025.2	9,86	2,27	4,81	4,81	0,72	1,28E-04	4,85E-04
AC005041.1	7,74	-2,68	0,16	-6,39	0,85	1,62E-04	6,06E-04
FGF21	3,71	5,02	32,43	32,43	2,49	1,72E-04	6,39E-04
VGLL1	9,32	-2,29	0,20	-4,88	0,75	1,76E-04	6,54E-04
CTNNA3	9,20	-2,26	0,21	-4,79	0,74	1,80E-04	6,67E-04
KRT8P39	9,04	2,34	5,06	5,06	0,77	2,10E-04	7,69E-04
AC012368.1	6,93	3,65	12,59	12,59	1,11	2,29E-04	8,33E-04
SLC34A3	6,98	-2,81	0,14	-7,01	0,93	2,46E-04	8,90E-04
AL121938.1	5,48	4,04	16,40	16,40	1,40	2,65E-04	9,55E-04
AC100860.1	9,34	-2,24	0,21	-4,74	0,78	2,83E-04	1,01E-03
DRP2	9,73	2,07	4,20	4,20	0,71	2,94E-04	1,05E-03
ASCL4	9,48	-2,06	0,24	-4,16	0,73	3,16E-04	1,12E-03
AP006287.2	9,32	-2,08	0,24	-4,22	0,73	3,37E-04	1,19E-03
REG4	5,14	-3,96	0,06	-15,60	1,45	3,54E-04	1,24E-03
HLA-DRB6	8,98	-2,17	0,22	-4,49	0,77	3,58E-04	1,26E-03
DPEP2	7,78	-2,38	0,19	-5,21	0,84	3,59E-04	1,26E-03
ANXA1	7,51	2,52	5,75	5,75	0,88	3,79E-04	1,32E-03
SEMA6D	4,89	-3,91	0,07	-14,99	1,44	3,91E-04	1,36E-03
Z73420.1	7,00	2,66	6,32	6,32	0,93	3,91E-04	1,36E-03
CPXM1	3,29	4,66	25,26	25,26	2,51	4,10E-04	1,42E-03
THORLNC	7,01	2,68	6,42	6,42	0,95	4,38E-04	1,51E-03
AC126696.1	6,92	2,65	6,28	6,28	0,94	4,49E-04	1,54E-03
ADAMTS18	5,96	-3,47	0,09	-11,08	1,16	4,66E-04	1,60E-03
CALHM5	7,59	-2,29	0,20	-4,89	0,85	4,92E-04	1,68E-03
Z82198.2	3,02	-4,61	0,04	-24,44	2,57	5,09E-04	1,73E-03
COL6A4P2	5,79	-3,41	0,09	-10,63	1,16	5,62E-04	1,90E-03
FLRT1	3,00	4,44	21,69	21,69	2,53	6,56E-04	2,19E-03
AC005703.5	5,79	-2,86	0,14	-7,26	1,06	7,14E-04	2,36E-03
MIR924HG	8,27	2,09	4,25	4,25	0,81	7,29E-04	2,41E-03
AC008514.1	2,71	-4,33	0,05	-20,17	2,60	8,88E-04	2,89E-03
TRIM55	6,65	2,52	5,74	5,74	0,99	8,93E-04	2,90E-03
IL1RAPL2	7,21	-2,22	0,21	-4,67	0,89	9,21E-04	2,98E-03
FAR2P3	5,31	-3,23	0,11	-9,39	1,18	9,21E-04	2,98E-03
AC061961.1	6,75	-2,32	0,20	-4,98	0,94	9,56E-04	3,08E-03
AC078942.1	6,52	-2,29	0,20	-4,90	0,93	1,00E-03	3,21E-03

BPIFA2	5,37	-3,16	0,11	-8,96	1,21	1,05E-03	3,35E-03
MUC16	4,28	-3,53	0,09	-11,57	1,53	1,08E-03	3,45E-03
MIR616	6,73	2,21	4,62	4,62	0,92	1,21E-03	3,81E-03
LURAP1L-AS1	2,67	4,09	17,06	17,06	2,58	1,24E-03	3,91E-03
NFE2	6,94	2,17	4,50	4,50	0,94	1,34E-03	4,20E-03
TIMP4	4,11	3,40	10,54	10,54	1,50	1,35E-03	4,22E-03
LINC01768	5,38	-2,67	0,16	-6,35	1,12	1,45E-03	4,52E-03
B3GNT6	2,60	3,98	15,80	15,80	2,63	1,58E-03	4,88E-03
AC010442.2	5,21	-3,08	0,12	-8,46	1,27	1,64E-03	5,05E-03
MIR4482	2,52	3,91	15,03	15,03	2,62	1,65E-03	5,07E-03
EREG	5,36	2,98	7,88	7,88	1,24	1,68E-03	5,15E-03
SCGN	2,44	-3,96	0,06	-15,54	2,68	1,69E-03	5,19E-03
SEPTIN5	5,04	2,98	7,88	7,88	1,21	1,73E-03	5,29E-03
AC137834.2	2,49	3,87	14,63	14,63	2,63	1,77E-03	5,40E-03
KLHDC7B-DT	5,04	2,92	7,54	7,54	1,23	1,91E-03	5,80E-03
LSMEM2	4,92	2,92	7,57	7,57	1,22	1,98E-03	5,98E-03
AL121949.1	2,51	3,79	13,85	13,85	2,68	2,02E-03	6,09E-03
AC138965.1	5,48	-2,20	0,22	-4,58	1,03	2,22E-03	6,64E-03
AC004877.2	5,95	-2,07	0,24	-4,18	0,98	2,27E-03	6,79E-03
GRAMD4P7	2,27	-3,70	0,08	-13,00	2,76	2,41E-03	7,17E-03
AL121888.1	4,81	-2,41	0,19	-5,33	1,14	2,67E-03	7,87E-03
AC105046.1	6,20	2,01	4,03	4,03	0,99	2,72E-03	7,99E-03
AL133467.1	2,30	3,56	11,77	11,77	2,76	2,78E-03	8,17E-03
TBC1D26-AS1	2,26	3,49	11,23	11,23	2,75	2,87E-03	8,40E-03
AC106886.3	3,56	3,02	8,10	8,10	1,60	2,97E-03	8,67E-03
SLC22A1	4,82	-2,34	0,20	-5,08	1,16	3,02E-03	8,80E-03
CCDC144NL-AS1	3,52	2,92	7,56	7,56	1,59	3,03E-03	8,81E-03
AC002310.2	3,56	2,94	7,68	7,68	1,61	3,05E-03	8,86E-03
LINC00867	3,39	-2,98	0,13	-7,91	1,64	3,10E-03	8,98E-03
HLX	4,97	2,34	5,05	5,05	1,16	3,27E-03	9,44E-03
MPPED1	2,19	3,38	10,44	10,44	2,81	3,28E-03	9,46E-03
