# Supplementary material for: CEBP-β and PLK1 as Potential Mediators of the Breast Cancer/Obesity Crosstalk: In Vitro and In Silico Analyses
Source: Nutrients. 2023 Jun 22;15(13):2839. doi: 10.3390/nu15132839 (PMC10343266; doi:10.3390/nu15132839)
Supplement: Supplementary file 1 [file nutrients-15-02839-s001.zip › nutrients-2438000-supplementary/Supplementary Table S3.pdf]

**Supplementary Table S3.** Twenty-eight of the 364 deregulated genes (FDR ⩾ 0.01 and fold change -2 ⩾ log2FC ⩾ 2) between CM untreated (control) and CM treated (CMT) MCF-7 BC cells.

| | MetaCore net symbol | MetaCore Name | Localizations | Edges |
|---|---|---|---|---|
| 1 | | AHRR | nucleus | 1 |
| 2 | | Amphiregulin | extracellular region | 0 |
| 3 | | Aquaporin 3 | membrane | 0 |
| 4 | | C/EBPbeta | nucleus | 6 |
| 5 | | C5aR | membrane | 0 |
| 6 | | Cacng6 | membrane | 0 |
| 7 | | CADPS | cytoplasm | 0 |
| 8 | | Calmegin | cytoplasm | 0 |
| 9 | | CEBPG | nucleus | 0 |
| 10 | | CEP3 | cytoplasm | 0 |
| 11 | | Ceruloplasmin | extracellular region | 1 |
| 12 | | Collagen II | extracellular region | 1 |
| 13 | | CYP1A1 | cytoplasm | 2 |
| 14 | | CYP4F3 | cytoplasm | 0 |
| 15 | | ENPP2 | extracellular region | 0 |
| 16 | | EPAS1 | nucleus | 4 |
| 17 | | Epiregulin | extracellular region | 1 |
| 18 | | GluR7 | membrane | 0 |
| 19 | | IBP5 | extracellular region | 1 |
| 20 | | IL-1RI | membrane | 0 |
| 21 | | ITGB6 | membrane | 0 |
| 22 | | PDE11A | cytoplasm | 0 |
| 23 | | PTG | cytoplasm | 1 |
| 24 | | SLC3A2 | membrane | 1 |
| 25 | | SLC7A5 | membrane | 2 |
| 26 | | SOCS2 | cytoplasm | 0 |
| 27 | | VEGFR-2 | membrane | 1 |
| 28 | | WNT3A | extracellular region | 0 |
