# Supplementary figures and images for: CEBP-β and PLK1 as Potential Mediators of the Breast Cancer/Obesity Crosstalk: In Vitro and In Silico Analyses
Source: Nutrients. 2023 Jun 22;15(13):2839. doi: 10.3390/nu15132839 (PMC10343266; doi:10.3390/nu15132839)

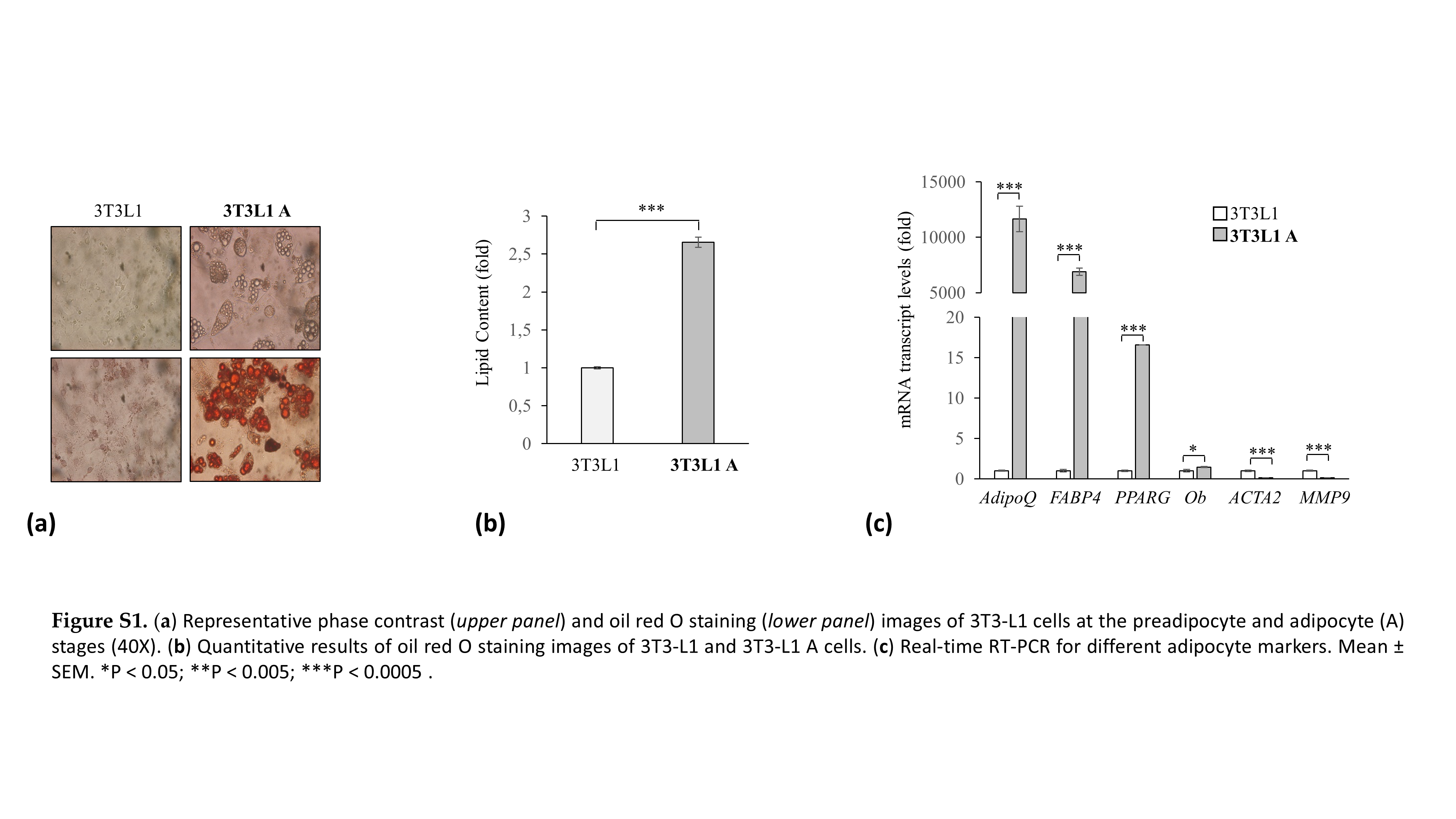

Supplement: Supplementary file 1 [file nutrients-15-02839-s001.zip › nutrients-2438000-supplementary/Supplementary Figure S1.tif]

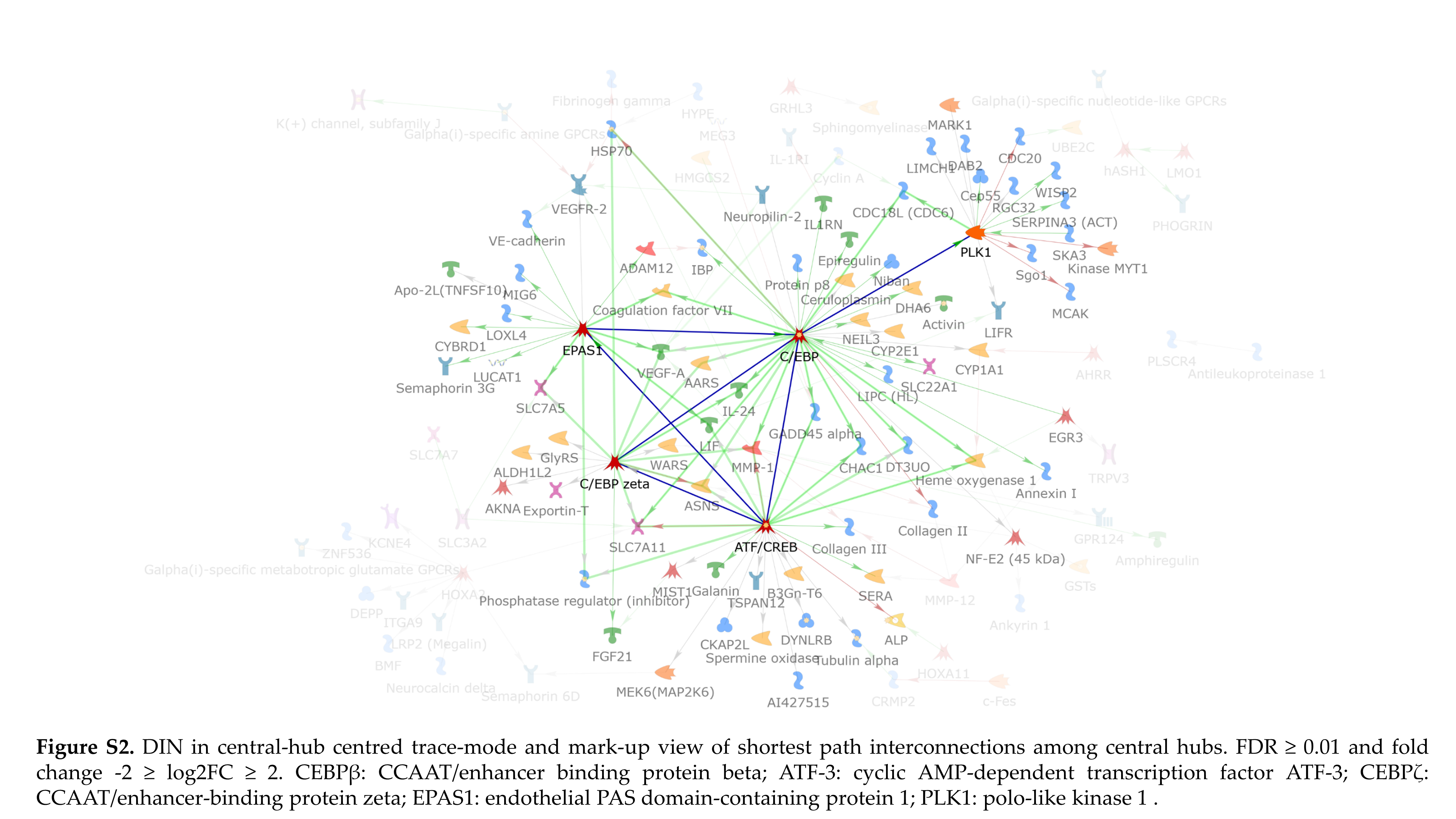

Supplement: Supplementary file 1 [file nutrients-15-02839-s001.zip › nutrients-2438000-supplementary/Supplementary Figure S2.tif]

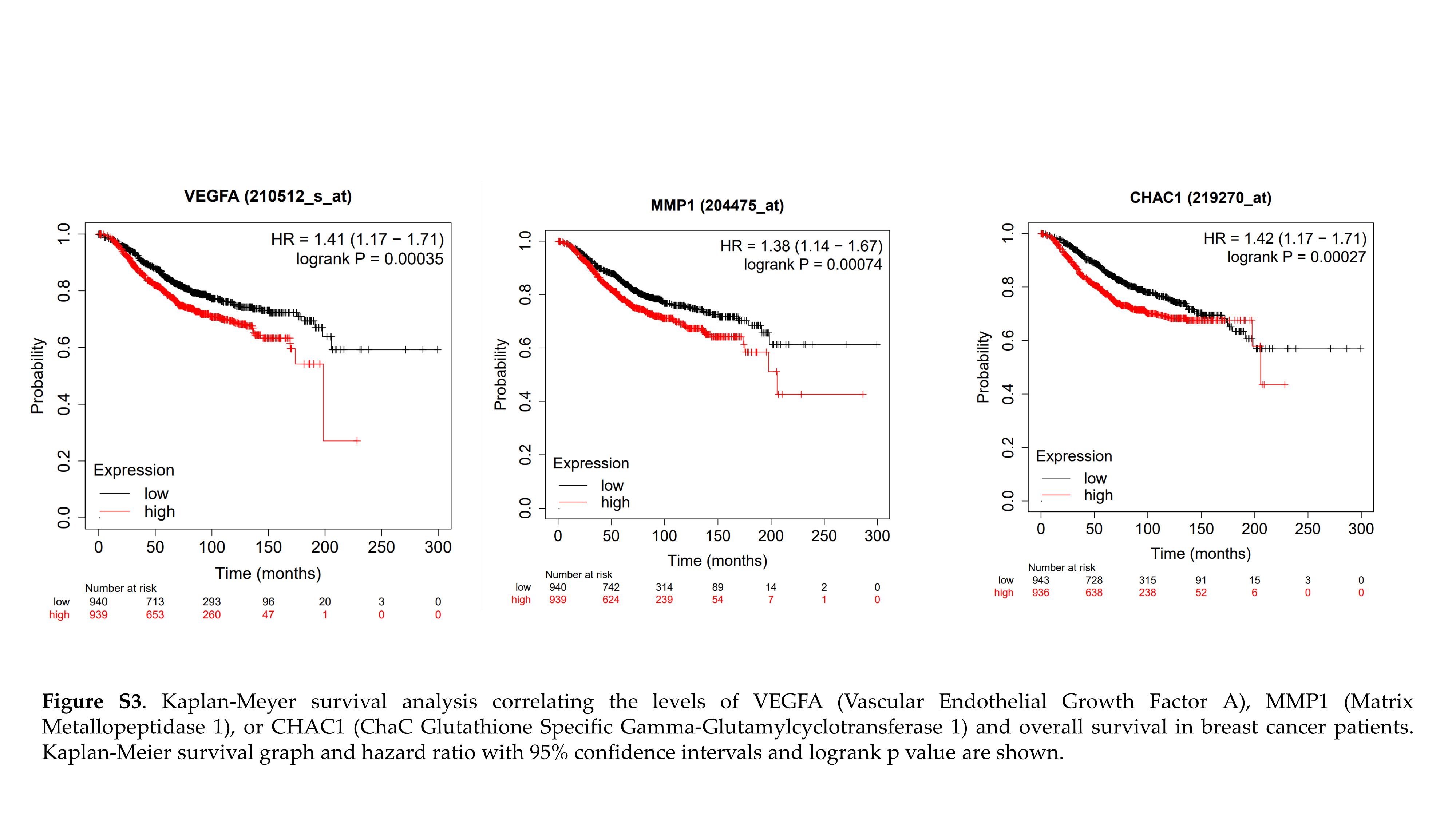

Supplement: Supplementary file 1 [file nutrients-15-02839-s001.zip › nutrients-2438000-supplementary/Supplementary Figure S3.tif]

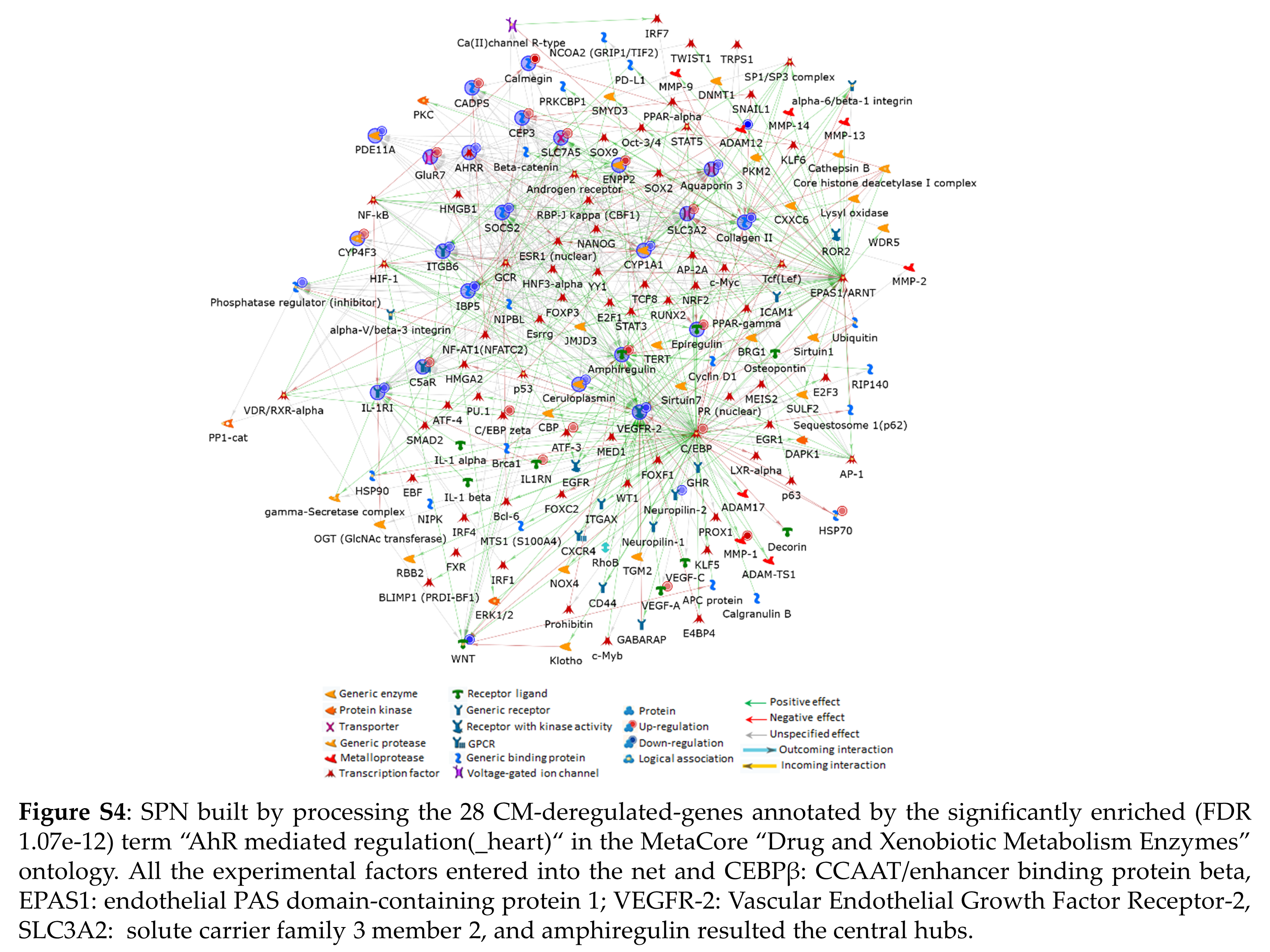

Supplement: Supplementary file 1 [file nutrients-15-02839-s001.zip › nutrients-2438000-supplementary/Supplementary Figure S4.tif]
